# Human leukocyte antigen genotype modulates cytomegalovirus- and Epstein–Barr virus-specific T-cell response in healthy donors for optimized virus-specific T-cell donor selection

**DOI:** 10.3389/fimmu.2026.1856550

**Published:** 2026-08-14

**Authors:** Rut Mora-Buch, Helena Pasamar, Maria Tomás-Marín, Emma Enrich, María Andrés-Rozas, Cleofé Peña-Gómez, Miriam García Bosca, Francesc Rudilla

**Affiliations:** 1 Advanced & Cell Therapy Services, Blood and Tissue Bank (BST), Barcelona, Spain; 2 Transfusional Medicine Group, Vall d'Hebron Research Institute, Universitat Autònoma de Barcelona (VHIR-UAB), Barcelona, Spain; 3 Immunogenetics and Histocompatibility Laboratory, Blood and Tissue Bank (BST), Barcelona, Spain; 4 Barcelona Supercomputing Center, Barcelona, Spain

**Keywords:** allogenic immunotherapy, CMV, EBV, HLA, virus-specific T cells

## Abstract

**Introduction:**

Reactivation of cytomegalovirus (CMV) and Epstein–Barr virus (EBV) is a major complication in immunocompromised patients, and understanding donor variability in antiviral immunity is essential for optimizing adoptive T-cell therapy. This study aimed to identify immunogenetic determinants of virus-specific T-cell responses in healthy donors to guide donor selection.

**Methods:**

CMV- and EBV-specific T-cell responses were characterized using flow cytometry and IFN-g ELISpot after stimulation with peptide pools, followed by integrated analyses correlating the response magnitude with human leukocyte antigen (HLA) class I and II alleles, HLA haplotypes, and HLA-G 3′UTR polymorphisms.

**Results:**

CMV 65-kDa phosphoprotein (pp65) induced stronger CD8^+^ and CD4^+^ T-cell responses than immediate early 1 (IE1), whereas the EBV consensus peptides preferentially activated CD8^+^ T cells. Several HLA alleles and linked haplotypic backgrounds were associated with enhanced or reduced CMV-specific T-cell responses, including an A*01:01~C*07:01~B*08:01~DRB1*03:01~DQB1*02:01 haplotype enriched among low responders. For EBV, several HLA-restricted alleles were associated with stronger peptide-specific activation, supporting the broad immunogenicity of the consensus peptide pool. Moreover, HLA-G 3′UTR polymorphisms selectively modulated the CMV-specific IFN-γ^+^ CD8^+^ T-cell frequencies.

**Discussion:**

Together, these results indicate that classical and non-classical HLA variations are associated with inter-donor differences in antiviral T-cell response in an antigen- and virus-dependent manner, providing a translational framework to refine donor selection and improve the design of T-cell-based immunotherapies.

## 1 Introduction

Allogeneic virus-specific T-cell (VST) therapy is an effective and safe strategy for the prevention and treatment of infections caused by latent herpesviruses such as cytomegalovirus (CMV) and Epstein–Barr virus (EBV) in immunocompromised patients (1–5). One of the main challenges for this therapy is the genetic diversity of the human leukocyte antigen (HLA) system, which influences antigen presentation and the magnitude of antiviral T-cell response. Accurate donor HLA characterization can therefore inform the selection and functional assessment of cell therapy candidates (6, 7).

Several studies have focused on the T-cell response to specific viral peptides presented by defined HLA molecules, providing important insights into the immune recognition mechanisms against viruses (8–10). However, this targeted approach does not fully reflect the context of VST production, where donor availability, HLA-matched donor selection, and Good Manufacturing Practice (GMP)-compliant protocols often require the use of commercially available antigen preparations. For CMV, whole viral protein peptide pools such as the 65-kDa phosphoprotein (pp65) and immediate early 1 (IE1) are commonly employed, whereas for EBV, GMP-grade pools of immunodominant peptides presented by prevalent HLA alleles may be applied. These approaches are not customized for the less common HLA alleles and do not fully address the inherent complexity of HLA-mediated responses, including the HLA haplotype donor heterozygosity, HLA allele linkage disequilibrium (LD), haplotypic effects, or the potential intra-individual immunodominance among presented epitopes. These factors influence the inter-donor variability in antiviral immunity across donors and may therefore be relevant for the generation of polyclonal VST products and their subsequent clinical effectiveness and safety.

This study investigated the associations between HLA genetic variation and CMV- or EBV-specific T-cell response in a large cohort of healthy individuals included in a cell donor registry (7). Using IFN-γ ELISpot assays in parallel with flow cytometric analysis, we confirm that ELISpot reflected the antigen-induced T-cell-driven response under the experimental conditions tested. Because donors were HLA-heterozygous and stimulated with multi-epitope peptide pools, this study was not designed to determine the independent molecular contribution of the individual HLA alleles or to define exact peptide–HLA restrictions. Instead, we applied multiple complementary statistical and stratification approaches to identify the immunogenetic patterns associated with stronger or weaker antigen-specific responses in a real-world donor screening context. Association analyses identified HLA alleles, linked haplotypic backgrounds, and HLA-G 3′UTR polymorphisms associated with differential CMV- and EBV-specific T-cell responses, providing a framework for improving VST donor prioritization and registry annotation.

## 2 Materials and methods

### 2.1 Study participants

A total of 203 healthy donors enrolled in the ReDoCel registry approved by the Ethics Committee of Vall d’Hebron University Hospital [Project PR (BST) 530/2018; protocol code: RTC-2017-6368-1] (7, 11, 12) were included (**Table 1**; **Supplementary Table S1**).

**Table 1:** Demographic characteristics of the cell donors analyzed by IFN-g enzyme-linked immunospot (ELISpot).

	Donors	Sex: F/M	Blood group: A/B/O/AB	Rh +/-/null	Age (years)	CMV IgG (+/-)	EBV IgG (+/-)	ELISpot: R/NR
Total	203	115/88	94/9/97/3	153/49/1	29 (20-57)	170/33	197/6	
CMV pp65	200	113/87	92/9/96/3	150/49/1	30 (20-57)	165/35	190/10	164/36
CMV IE1	176	99/77	83/8/83/2	133/43/0	29 (20-57)	147/29	170/6	103/67
EBV	189	106/83	86/8/92/3	144/44/1	29 (20-57)	159/30	183/6	176/13
Stats.		ns	ns	ns	ns	ns	ns	*p* < 0.0001

### 2.2 HLA frequencies and linkage disequilibrium

The HLA-net GENE[RATE] pipeline (13) was used to calculate the HLA allele and haplotype frequencies and LD analysis. The HLA allele frequencies and LD were analyzed for the HLA-A, HLA-B, HLA-C, HLA-DRB1, HLA-DQB1, HLA-DQA1, HLA-DPB1, HLA-DPA1, HLA-E, and HLA-G loci at two-field resolution. Significant LD was considered when the standard residual values (stdres) were greater than 1.96. Haplotype frequencies were calculated for the *HLA-A*, *HLA-B*, *HLA-C*, *HLA-DRB1*, and *HLA-DQB1* genes.

### 2.3 PBMC stimulation with peptide pools

As previously described (12), thawed peripheral blood mononuclear cells (PBMCs) were cultured and stimulated with PepTivator peptide pools (Miltenyi Biotec, Bergisch Gladbach, Germany) at 50 ng/ml in the presence of interleukin 2 (IL-2) at 50 U/ml (Miltenyi Biotec). Cells were cultured at 2 × 10^6^ cells/ml for 6 days in complete medium (CM) consisting of RPMI-1640 (Gibco, Waltham, MA, USA) supplemented with 10% human AB serum (hSerAB; Banc de Sang i Teixits, Barcelona, Spain) at 37°C in a 5% CO_2_ atmosphere. The CMV pp65 and IE1 PepTivator peptide pools contained 15-mer sequences with an 11-amino acid overlap, covering the full length of the CMV pp65 or CMV IE1 protein. In contrast, the EBV consensus PepTivator consisted of 43 selected peptides of 8–20 amino acids derived from 13 EBV proteins, including immunodominant epitopes with annotated HLA restrictions. Therefore, the CMV pp65 and IE1 responses were analyzed as responses to overlapping whole-protein peptide pools, whereas the EBV responses were interpreted as responses to this specific GMP-grade consensus peptide pool rather than as a comprehensive measure of the total EBV-specific immunity. PBMCs cultured for 6 days with IL-2 alone were used as negative controls for the enzyme-linked immunoSpot (ELISpot) and flow cytometry stimulation assays.

### 2.4 IFN-γ ELISpot assay

On day 6, cells were re-stimulated with the same PepTivators at 50 ng/ml. Peptide-stimulated cells were cultured overnight in duplicate at 2.5 × 10^4^ cells/well in IFN-γ ELISpot plates using the Human IFN-γ ELISpot^PRO^ Kit (Mabtech, Stockholm, Sweden) at 37°C and 5% CO_2_. The ELISpot assay was performed according to the manufacturer’s instructions and analyzed as previously reported (12). Donors were categorized into two groups: responders (R), defined as those with peptide pool IFN-γ spot forming unit (SFU) values ≥30 and at least twice the negative control, or non-responders (NR), those who did not meet one of the R criteria. Negative control wells consisted of cells cultured under identical conditions in the absence of viral antigen. Donor-specific background was therefore incorporated into the responder definition by requiring the antigen-stimulated wells to reach both an absolute threshold of ≥30 SFU and at least a twofold increase over the corresponding negative control. In addition, background-adjusted SFU values were calculated by subtracting the paired negative control SFU from the antigen-stimulated SFU for each donor and were used in the sensitivity analyses to confirm that the main associations were not driven by elevated background signal. R donors were divided into three groups for each antigen based on the quartile (Q) of the values: high R (HR), with IFN-γ SFU ≥Q3; medium R (MR), with IFN-γ SFU <Q3 to ≥Q1; or low R (LR), with IFN-γ SFU <Q1 to >30 SFU.

### 2.5 T-cell stimulation and flow cytometry staining

On day 6, the cells were re-stimulated with the same PepTivator peptide pools (Miltenyi Biotec) per condition at 1 µg/ml and cytokine release inhibitors for 5 h with a-CD107a (H4A3) in the culture. For flow cytometry staining, the cells were incubated with ViaKrome fixable viability dye (Beckman Coulter, Pasadena, CA, USA) diluted 1:200 for 10 min at room temperature (RT). Subsequently, antibodies directed against surface markers CD3 (UCHT1), CD4 (13B8.2), CD8 (B9.11), CD56 (N901) (Beckman Coulter), CD45RA (HI100), and CCR7 (G043H7) (BioLegend, San Diego, CA, USA) were added and incubated for 20 min at RT. Thereafter, intracellular staining was conducted following the manufacturer’s instructions in the Fixation/Permeabilization Kit (BD Biosciences, San Jose, CA, USA) with antibodies against the intracellular antigens interferon gamma (IFN-γ) (45.15) and tumor necrosis factor (TNF) (IPM2) (Beckman Coulter). Stained samples were acquired using a MACSQuant (Miltenyi Biotec) or a CytoFLEX (Beckman Coulter) flow cytometer. TNF and CD107a were included as exploratory functional markers of antigen-induced T-cell activation and potential polyfunctionality. However, IFN-γ was selected as the primary functional readout for this study because IFN-γ ELISpot was performed in the full donor cohort and was used for the donor classification and HLA association analyses. The TNF or CD107a data were therefore not used for the primary immunogenetic analyses. The flow cytometry results were expressed as the percentage of cytokine- or T-cell memory marker-positive cells within gated CD3^+^, CD4^+^CD3^+^, or CD8^+^CD3^+^ T-cell populations.

### 2.6 Flow cytometry analysis and unsupervised clustering

The flow cytometry data were analyzed using FlowJo version 10. After the exclusion of debris, dead cells, and doublets, the lymphocytes were gated according to the forward- and side-scatter properties, followed by the identification of CD3^+^ T cells and subsequent gating of the CD4^+^CD3^+^ and CD8^+^CD3^+^ T-cell subsets (**Supplementary Figure S1**). The IFN-γ^+^ and IFN-γ^+^TNF^+^ gates were defined using isotype controls. CCR7 and CD45RA expression was used to describe the differentiation phenotypes after the 6-day antigen-driven culture, defining CCR7^+^CD45RA^+^ cells as naive-like, CCR7^+^CD45RA^−^ cells as central memory-like, CCR7^−^CD45RA^−^ cells as effector memory-like, and CCR7^−^CD45RA^+^ cells as terminally differentiated effector memory (TEMRA)-like. Because cells had undergone antigen stimulation and short-term culture before phenotypic analysis, these categories were interpreted as culture-associated CCR7/CD45RA-defined phenotypes rather than definitive *ex vivo* memory subsets. For the clustering analysis, compensated flow cytometry files were preprocessed by applying the same quality control and lineage gates described above. The IFN-γ^+^TNF^+^ cells from each sample were gated and concatenated. t-Distributed stochastic neighbor embedding (t-SNE) analysis was performed for dimensionality reduction and unsupervised clustering with the X-shift plugin.

### 2.7 Statistics

GraphPad Prism 5 (GraphPad, San Diego, CA, USA) or Python version 3.9.16 was used to generate graphs and perform statistical analyses. Data normality was assessed using the Shapiro–Wilk test. For all analyses, *p-*values less than 0.05 were considered statistically significant, as indicated by one or more asterisks or a dash. For two-group analysis, multiple-group comparisons, and correlation analysis, the Mann–Whitney test, Kruskal–Wallis test followed by Dunn’s multiple-group comparison *post-hoc* test, and Spearman’s correlation were used, respectively. For paired analysis, Wilcoxon matched-pairs test was used. Bootstrap analyses were used as complementary exploratory analyses to assess the stability of associations in the context of imbalanced group sizes. For bootstrap comparison, we first calculated the observed *U* statistic between the target group and the rest of the sample. The two sets of values were then pooled, and 5,000 bootstrap resamples were generated by repeatedly drawing, with replacement, two new samples of the same sizes as the original target and the remaining groups. For each bootstrap iteration, a *U* statistic was computed, yielding an empirical null distribution. The bootstrap *p*-value was defined as the proportion of bootstrap *U* statistics whose absolute deviation from the empirical median was greater than or equal to that of the observed *U* statistic. For HLA allele association analyses, donors carrying a given allele were compared with donors not carrying that allele. Because donors were HLA-heterozygous and multiple linked HLA alleles may contribute to the response, these analyses were interpreted as allele presence association analyses rather than as tests of independent causal effects of individual HLA molecules. To account for multiple testing across HLA comparisons, *p*-values were adjusted using the Benjamini–Hochberg false discovery rate (FDR) method separately within each antigen-specific analysis and locus-stratified. Associations with adjusted *p*-values below 0.05 were considered statistically significant after multiple comparison correction, whereas nominal associations that did not remain significant after correction were considered exploratory. For selected HLA alleles associated with differential antigen-specific responses, exploratory allele copy number analyses were performed by stratifying donors as non-carriers, heterozygous carriers, or homozygous carriers. The response distributions among the three groups were compared using the Kruskal–Wallis test, followed, when appropriate, by Dunn’s multiple comparison test. To avoid overinterpretation of very small groups, only those alleles represented by at least three homozygous donors or showing a significant overall Kruskal–Wallis test were included in the supplementary analysis.

## 3 Results

### 3.1 CMV- and EBV-specific T-cell response in PBMCs of cell donors

**Figure 1:**
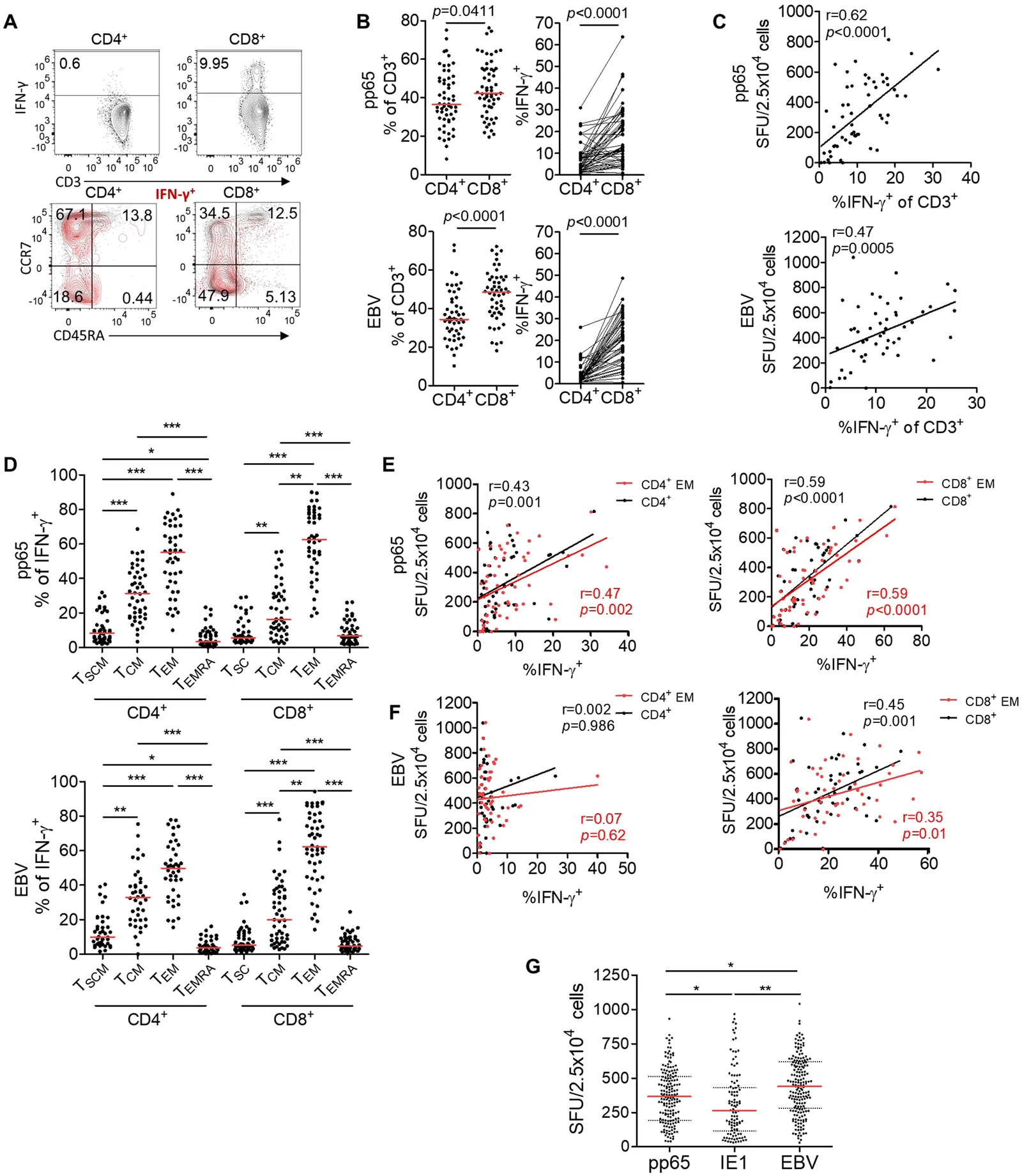
Thawed peripheral blood mononuclear cells (PBMCs) stimulated with cytomegalovirus (CMV) or Epstein–Barr virus (EBV) antigens. Detection of cell response by flow cytometry and IFN-γ ELISpot. **(A)** IFN-γ expression (in *red*) by CD4^+^CD3^+^ or CD8^+^CD3^+^ T cells and CD4^+^CD3^+^ or CD8^+^CD3^+^ T cells by CD45RA and CCR7 expression. **(B)** Left: Percentages of CD3^+^CD4^+^ and CD3^+^CD8^+^ T cells among CD3^+^ T cells of PBMCs stimulated with CMV pp65 (*n* = 57) or EBV (*n* = 51) peptide pools (Mann–Whitney test). Right: Percentages of IFN-γ^+^ cells among CD3^+^CD4^+^ or CD3^+^CD8^+^ cells T cells stimulated with CMV pp65 or EBV peptide pools (Wilcoxon matched-pairs test). **(C)** Correlations between spot forming units (SFUs) of IFN-γ ELISpot and percentages of IFN-γ^+^ among CD3^+^ T cells by flow cytometry after stimulation with the CMV pp65 peptide pool or with the EBV consensus peptide pool. **(D)** Percentages of cells expressing memory T-cell markers, stem cell memory (SCM), central memory (CM), effector memory (EM), and effector memory CD45RA^+^ (EMRA) among IFN-γ^+^ CD3^+^CD4^+^ and CD3^+^CD8^+^ T cells stimulated with CMV pp65 (*upper*) or the EBV peptide pool (*lower*) (Friedman test, posttest Dunn’s multiple comparison test). Because CCR7 and CD45RA expression was assessed after 6 days of antigen stimulation in the presence of IL-2, these phenotypes should be interpreted as culture-associated CCR7/CD45RA-defined differentiation states rather than definitive *ex vivo* memory subsets. **(E, F)** Correlations between SFUs of the IFN-γ ELISpot and the percentages of IFN-γ^+^ among CD4^+^CD3^+^ (in *black*) and CD45RA^−^CCR7^−^CD4^+^CD3^+^ (EM) (in *red*) T cells or CD3^+^CD8^+^ ( in *black*) and CD45RA^−^CCR7^−^CD8^+^CD3^+^ (EM) ( in *red*) T cells by flow cytometry stimulated with the CMV pp65 peptide pool (*n* = 51) **(E)** or with the EBV consensus peptide pool (*n* = 49) **(F)** (Spearman’s correlation). **(G)** SFU (negative control subtracted) of 2.5 × 10^4^ cells stimulated with viral peptide pools of healthy donors classified as responders (*R*), 164 donors with CMV pp65, 103 donors with CMV IE1, and 176 donors with EBV. High responders (*HR*) with IFN-γ SFU ≥quartile 3 (Q3), medium responders (*MR*) with IFN-γ SFU <Q3 to ≥Q1, or low responders (*LR*) with IFN-γ SFU <Q1 to >30 SFU. Pp65: Q1 = 209.3 and Q3 = 509.5; IE1: Q1 = 106 and Q3 = 459.8; EBV: Q1 = 272 and Q3 = 605. Kruskal–Wallis test followed by Dunn’s multiple comparison posttest. *p < 0.05, **p < 0.005, ***p < 0.0005.

To evaluate whether the IFN-γ ELISpot results represented T-cell response to the CMV or EBV antigens, we compared the ELISpot outcomes with the IFN-γ-producing cells measured by flow cytometry in healthy donors (**Supplementary Table S1**; **Figure 1A**; **Supplementary Figure S1**) (12). On day 6, CMV pp65 and EBV consensus PepTivators induced a higher proportion of CD8^+^CD3^+^ than CD4^+^CD3^+^ T cells, and the frequency of IFN-γ-producing CD8^+^CD3^+^ T cells was significantly higher than that of CD4^+^CD3^+^ T cells for both viral antigens upon re-stimulation (**Figure 1B**). To validate that the IFN-γ production detected by the ELISpot assay was attributable to the T-cell-mediated response, we performed correlation analyses between the ELISpot results and the IFN-γ production in the T-cell subsets using flow cytometry. A significant correlation was observed between the ELISpot results and the IFN-γ production by CD3^+^ T cells in PBMCs stimulated with either the CMV pp65 or the EBV peptide pool (**Figure 1C**). After 6 days of culture, as the major memory phenotype T-cell population among CD4^+^CD3^+^T or CD8^+^CD3^+^T are CCR7^−^CD45RA^−^ cells, classified as effector memory T cells (T_EM_) (**Figure 1D**), a significant correlation was found between SFU and IFN-γ production by the CD8^+^CD3^+^T and CD8^+^CD3^+^ T_EM_ cells stimulated with either the CMV pp65 or the EBV peptide pool (**Figures 1E, F**). In contrast, the SFU and IFN-γ production by CD4^+^CD3^+^T or CD4^+^CD3^+^ T_EM_ correlated only in the samples stimulated with CMV pp65 and not with the EBV peptides. This finding suggests that the ELISpot-detected IFN-γ response to the EBV consensus PepTivator was predominantly driven by CD8^+^ T cells. These data support the proposal that ELISpot data reliably represent the T-cell response to the tested antigens.

Although the ELISpot and flow cytometry results were correlated, the absolute values obtained with the two assays were not directly interchangeable. The ELISpot results were expressed as IFN-γ SFU per 25,000 total cultured cells after overnight re-stimulation with 50 ng/ml peptide pool. In contrast, flow cytometry quantified the percentage of IFN-γ-producing cells within the gated CD4^+^ or CD8^+^ T-cell subsets after 5 h of re-stimulation with 1 µg/ml peptide pool in the presence of cytokine secretion inhibitors. Therefore, high frequencies of IFN-γ^+^ cells within a selected T-cell subset may coexist with lower SFU values when expressed relative to the total plated cells. Flow cytometry was therefore used to identify the cellular source and subset distribution of IFN-γ production, whereas ELISpot was used as the scalable functional readout for donor classification and immunogenetic association analyses.

We examined a larger cohort of 205 healthy donors using ELISpot, whose PBMCs were stimulated with CMV pp65 (*n* = 200), EBV consensus peptides (*n* = 189), and an additional CMV protein, IE1 (*n* = 175) (**Table 1**; **Supplementary Figure S2**). No significant differences in sex, age, blood group, or CMV or EBV serostatus were found across the donor groups (**Table 1**). The response frequencies differed by antigen, with EBV eliciting the highest proportion of R (93.2%), followed by CMV pp65 (82%) and CMV IE1 (58.2%). Consistently, among R, EBV stimulation generated significantly higher SFU counts than the CMV antigens, and the pp65 responses exceeded those of IE1 (**Supplementary Figure S2**; **Figure 1G**). R were categorized as HR, MR, or LR based on the SFU distribution quartiles for each antigen (**Figure 1G**). This classification allowed for a comparative evaluation of the T-cell response strength, contextualizing individual SFU values relative to the distribution observed across the cohort.

### 3.2 CMV and EBV IFN-γ ELISpot results distributed by donor seropositivity

Analysis of potential associations between the IFN-γ ELISpot responses and the donor characteristics revealed that only blood group B donors exhibited reduced responses to EBV peptides compared with groups A and O (**Supplementary Figure S3**). However, this finding is based on a small number of group B individuals and requires confirmation in larger cohorts.

**Figure 2:**
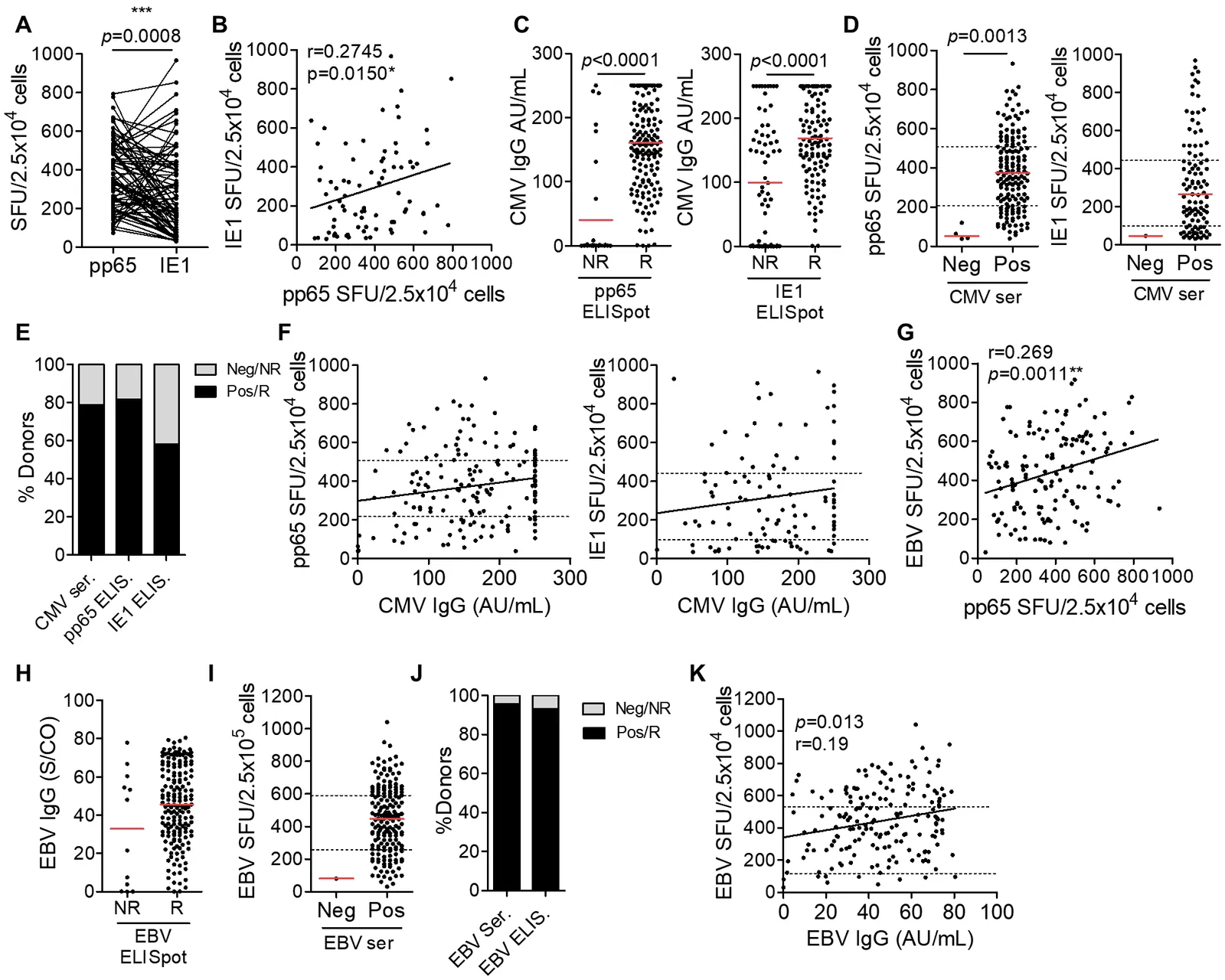
Thawed peripheral blood mononuclear cells (PBMCs) stimulated with cytomegalovirus (CMV) and Epstein–Barr virus (EBV) antigens. Detection of cell response by IFN-γ ELISpot. **(A)** IFN-γ spot forming unit (SFU) of 2.5 × 10^4^ cells stimulated with CMV proteins, pp65 and IE1, of 78 donors. Wilcoxon matched-pairs test. **(B)** Correlation of IFN-γ SFU between both CMV proteins, pp65 with IE1, of 78 donors. Spearman’s correlation. **(C)** Left: Immunoglobulin G (IgG) against CMV antigens detected in non-responder (*NR*, *n* = 33) or responder (*R*, *n* = 160) donors stimulated with pp65. Right: IgG against CMV antigens detected in NR (*n* = 70) or R (*n* = 120) donors stimulated with IE1. **(D)** Left: IFN-γ SFU of 2.5 × 10^4^ cells stimulated with pp65 in CMV-seropositive (*Pos*) or CMV-seronegative (*Neg*). Right: IFN-γ SFU of 2.5 × 10^4^ cells stimulated with IE1 in CMV-seropositive (*Pos*) or CMV-seronegative (*Neg*). **(E)** Percentage of donors with Neg or Pos serology against CMV antigens and percentage of NR or R donors in the IFN-γ ELISpot (*ELIS*.) stimulated with pp65 or IE1 peptide pools. **(F)** Left: Correlation of IFN-γ SFUCMV pp65 and IgG against CMV antigens detected in R (*n* = 164). Right: Correlation of IFN-γ SFU CMV IE1 and IgG against CMV antigens detected in R (*n* = 103). **(G)** Correlation of IFN-γ SFU CMV pp65 with EBV antigens of 145 donors. Spearman’s correlation. **(H)** IgG against EBV antigens detected in NR (*n* = 12) or R (*n* = 175) donors stimulated with EBV antigens. **(I)** IFN-γ SFU of 2.5 × 10^4^ cells stimulated with EBV antigens in EBV-seropositive (*Pos*) or EBV-seronegative (*Neg*). **(J)** Percentage of donors with Neg or Pos serology against EBV antigens and percentage of NR or R donors in the IFN-γ ELISpot (*ELIS*.) stimulated with EBV peptide pools. **(K)** Correlation of IFN-γ SFU EBV consensus and IgG against EBV antigens detected in R (*n* = 175). Spearman’s correlation. Median marked in red.

Paired analysis revealed lower SFU counts in response to IE1 *versus* pp65 (**Figure 2A**), although the response to both antigens correlated weakly, but significantly (**Figure 2B**). Donors classified as R to either pp65 or IE1 in the ELISpot assay showed significantly higher levels of CMV-specific immunoglobulin G (IgG) in serum compared with NR (**Figure 2C**). Notably, four CMV-seronegative donors were classified as LR, with significantly lower SFU counts than seropositive R (**Figure 2D**). The response rates to pp65 closely matched CMV seropositivity, whereas the IE1 responses were lower despite similar serostatus distribution (**Figure 2E**). Nonetheless, no significant correlation was observed between the ELISpot magnitude and the CMV IgG titers (**Figure 2F**), suggesting that the strength of the T-cell response does not directly reflect humoral immune status, or the assay detection boundaries could influence the observed discordance.

Comparison of the SFU counts to the CMV pp65 and EBV antigens showed a weak but significant correlation (**Figure 2G**). In contrast to CMV, the EBV-specific IgG levels did not differ between R and NR (**Figure 2H**), and one NR was EBV-seronegative (**Figure 2I**). Overall, the proportions of donors classified as R by either serology or ELISpot against EBV antigens were comparable and exceeded 93% of the cohort studied (**Figure 2J**). Furthermore, a significant but weak correlation was found between the SFU counts to EBV antigen stimulation and corresponding EBV-specific IgG levels (**Figure 2K**).

### 3.3 Association of HLA alleles with the antigen-specific T-cell response to the CMV and EBV peptide pools

**Figure 3:**
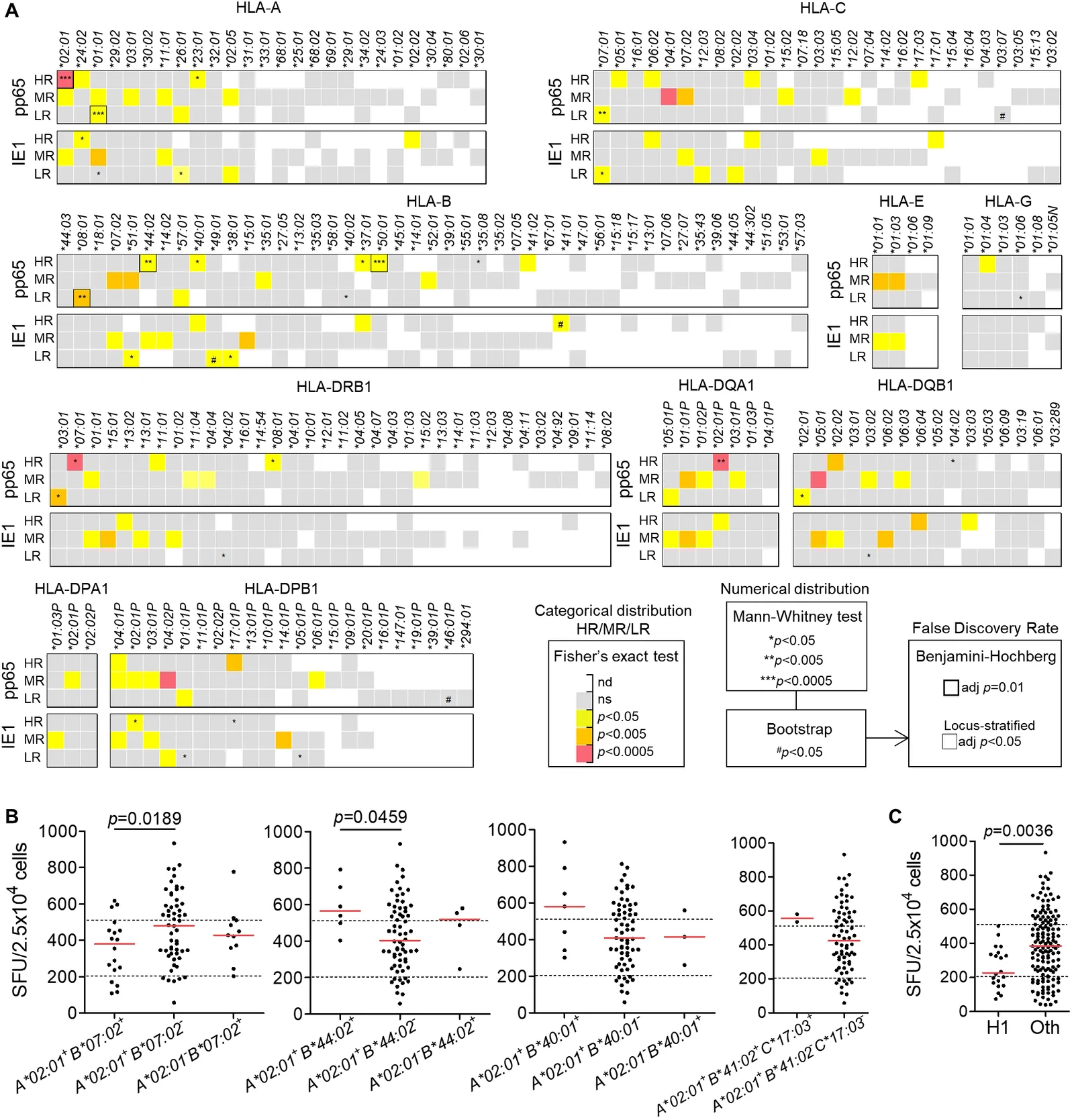
T-cell response of peripheral blood mononuclear cells (PBMCs) stimulated with cytomegalovirus (CMV) antigens distributed by the donor’s human leukocyte antigen (HLA). **(A)** The *X*-axis of the matrices represents the HLA distribution by frequency within the group of responder donors, while the *Y*-axis lists the donors distributed by categorical analysis as high responders (*HR*) with IFN-γ spot forming unit (SFU) ≥quartile 3 (Q3), medium responders (*MR*) with IFN-γ SFU <Q3 to ≥Q1, or low responders (*LR*) with IFN-γ SFU <Q1 to >30 SFU tested by viral antigens in the IFN-γ ELISpot assay: 164 for CMV pp65 and 103 for CMV IE1. Categorical distribution analyzed by Fisher’s exact test. *White* indicates no data (*nd*), *gray* indicates non-significant (*ns*) results, *yellow* indicates *p* < 0.05, *orange* indicates *p* < 0.005, and *red* indicates *p* < 0.0005 statistical significance. Numerical statistical differences in SFU per cell by ELISpot for donors with specific HLA compared to SFU/cell from other donors were analyzed using the Mann–Whitney test (*p < 0.05, **p < 0.005, ***p < 0.0005) and bootstrap (^#^*p* < 0.05) comparison. *Asterisks* or *hashes in HR positions* indicate significant numerical results of a higher median value of the specific HLA allele compared with median value of other donors. *Asterisks* or *hashes in LR positions* indicate significant numerical results of a lower median value of the specific HLA allele compared with the median value of the other donors. For false discovery rate, Benjamini–Hochberg significant adjusted *p*-values from the bootstrap *p*-values are marked as *framed box*. Associations that did not remain significant after multiple comparison correction should be interpreted as exploratory. Supporting data for this figure can be found in **Supplementary Figure S4** and **Supplementary Tables S6**, **S7**, **S9**. **(B)** IFN-γ SFU of 2.5 × 10^4^ cells stimulated with CMV pp65. **(C)** IFN-γ SFU of 2.5 × 10^4^ cells stimulated with CMV pp65 distributed by donors with the H1 haplotype (Mann–Whitney test or *t*-test). Median marked in *red*.

**Figure 4:**
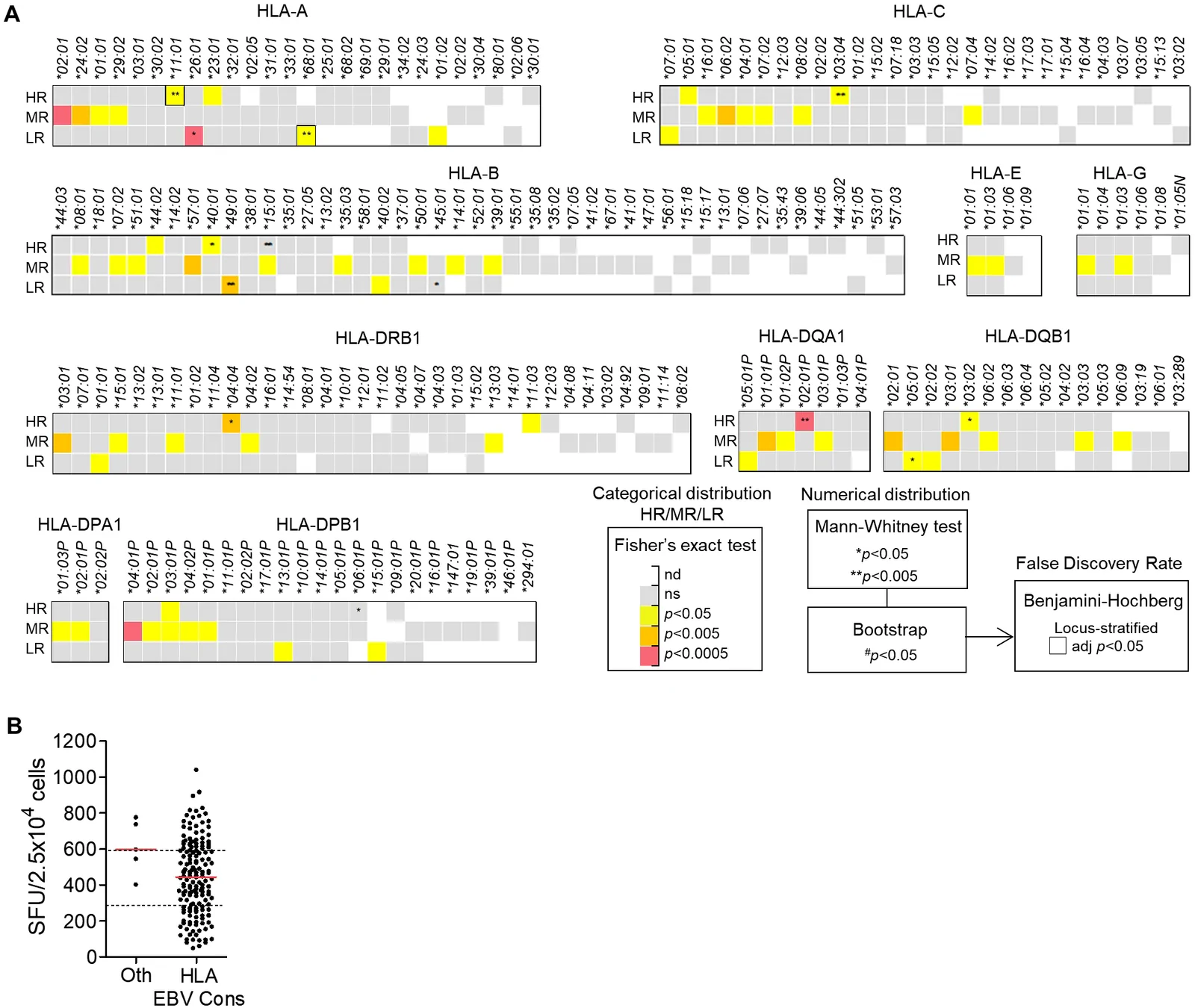
T-cell response of peripheral blood mononuclear cells (PBMCs) stimulated with Epstein–Barr virus (EBV) antigens distributed by donors’ human leukocyte antigen (HLA). **(A)** The *X*-axis of the matrices represents the HLA distribution by frequency within the group of responder donors, while the *Y*-axis list the 189 donors tested with EBV antigens in the IFN-γ ELISpot distributed by categorical analysis as high responders (HR) with IFN-γ spot forming unit (SFU) ≥quartile 3 (Q3), medium responders (*MR*) with IFN-γ SFU <Q3 to ≥Q1, or low responders (*LR*) with IFN-γ SFU <Q1 to >30 SFU. Categorical distribution analyzed by Fisher’s exact test. White indicates no data (nd), gray indicates non-significant (*ns*) results, yellow as *p* < 0.05, orange as *p* < 0.005, and red as *p* < 0.0005 statistical significance. Numerical statistical differences in spot SFUs per cell by ELISpot for donors with specific HLA compared with SFU/cell from other donors were analyzed using the Mann–Whitney test (*p < 0.05, **p < 0.005) and bootstrap (^#^*p* < 0.05) comparison. Asterisks in HR positions indicate significant numerical results of a higher median value of the specific HLA allele compared with the median value of other donors. Asterisks in LR positions indicate significant numerical results of a lower median value of the specific HLA allele compared with the median value of the other donors. For false discovery rate, Benjamini–Hochberg significant adjusted *p*-values from the bootstrap *p*-values are marked as framed box. Associations that did not remain significant after multiple comparison correction should be interpreted as exploratory. Supporting analysis for this figure can be found in **Supplementary Figure S4**; **Supplementary Tables S8**, **S9**. **(B)** IFN-γ SFU of 2.5 × 10^4^ cells stimulated with the EBV consensus PepTivator distributed by donors with HLA genes described in the PepTivator HLA molecule restriction and donors with HLA genes not included in the PepTivator restriction (*Oth*) (Mann–Whitney test or *t*-test). Median marked in red.

The HLA frequencies of the donors included in the study (**Supplementary Table S2**) closely aligned with those documented in the Spanish population (14). Given that the cell donors were heterozygous for HLA genes and the peptide pools used for stimulation were not restricted to a single HLA allele, we explored the associations between the presence of individual HLA alleles and antigen-specific T-cell response. These analyses were not intended to define the independent contribution of individual HLA molecules because multiple HLA class I and class II alleles may simultaneously contribute to peptide presentation and because HLA alleles occur in linked haplotypic backgrounds. The ELISpot results were analyzed based on the presence or absence of individual HLA alleles among R (**Supplementary Tables S3**–**S5**), and the results were interpreted as allele presence associations within the donor cohort. The results were evaluated both numerically, based on the SFU counts, and categorically, by stratifying donors into HR, MR, and LR for each antigen. Categorical differences were visualized by color coding (**Figures 3**, **4**; **Supplementary Tables S6**–**S8**), while statistically significant numerical differences were indicated by asterisks (**Figures 3**, **4**; **Supplementary Figure S4**). Notably, the categorical significance observed within the MR group reflected reduced response variability among donors carrying that specific HLA allele. Because certain allele combinations yielded very small target groups relative to the rest of the cohort, we complemented the standard analysis with a non-parametric bootstrap procedure to obtain a distribution-free estimate of significance that remained stable despite imbalanced group sizes. Bootstrap results validated the Mann–Whitney test findings and identified additional significant HLA alleles, marked with hashtags (**Figure 3**; **Supplementary Table S9**). To reduce the risk of false-positive findings due to the large number of HLA comparisons, *p*-values from the HLA association analyses were adjusted using the Benjamini–Hochberg FDR method within each antigen-specific dataset and locus-stratified. Associations that remained significant after correction are indicated as framed box, whereas nominally significant associations that did not survive correction could be considered as exploratory observations (**Figure 3**; **Supplementary Table S9**). Because several of the analyzed HLA alleles were in strong LD (**Supplementary Table S10**), the associations observed for linked alleles may represent a common haplotypic signal rather than independent effects.

### 3.4 Impact of HLA allelic variability on the CMV pp65- and IE1-specific T-cell response

Analysis of the CMV pp65-stimulated PBMCs identified HLA backgrounds associated with differential pp65-specific IFN-γ responses, including alleles previously reported to restrict immunodominant pp65 epitopes, such as HLA-A*02:01 (8, 15), HLA-DRB1*07:01, and DRB1*11:01 (9) (**Figure 3A**; **Supplementary Figure S4**; **Supplementary Table S6**). Donors carrying HLA-A*01:01, C*07:01, B*08:01, DRB1*03:01, or DQB1*02:01, which form the frequent haplotype H1, were enriched in the LR group following CMV pp65 stimulation (**Figure 3A**; **Supplementary Tables S10**, **S11**). Donors carrying the H1 haplotype showed a significantly lower response compared with the remaining donors (**Figure 3C**). CMV IE1 stimulation showed a similar pattern, with stronger responses in carriers of HLA-A*24:02, C*06:02, C*03:04, B*40:01, B*37:01, DQA1*02:01P, and DPB1*17:01P, while donors with A*01:01, and C*07:01 showed a reduced response (**Figure 3A**, **Supplementary Figure S4**; **Supplementary Tables S6**, **S7**).

HLA-A*02:01, the most frequent allele in our cohort, was associated with a strong CMV pp65-specific T-cell response (**Figure 3A**; **Supplementary Figure S4**). Although HLA-B*07:02 also restricts pp65 epitopes (8), it was enriched in the MR group, indicating limited variability. Donors carrying both A*02:01 and B*07:02 showed a reduced pp65 response compared with A*02:01-only individuals, suggesting that the magnitude of the pp65-specific response in A*02:01 carriers may be influenced by the broader class I HLA context (**Figure 3B**). In contrast, A*02:01 carriers with B*44:02 or B*40:01 exhibited higher responses, suggesting that additional class I HLA background may be associated with stronger pp65 reactivity in A*02:01-positive donors. HLA-B*41:02 and C*17:03, linked to A*02:01, were also found in HR donors (**Figure 3B**). Notably, 7 out of 10 A*02:01 LR donors carried at least one allele from the low-response haplotype H1, whereas only 3 out of 13 HR did (**Supplementary Table S11**).

Exploratory allele copy number analyses were performed for selected HLA alleles associated with differential antigen-specific responses. For several alleles, including A*02:01, A*01:01, C*07:01, DRB1*03:01, DQA1*05:01, DQB1*02:01, and DPB1*04:01, homozygous carriers exhibited higher median responses than did heterozygous carriers. However, the differences between heterozygous and homozygous donors were not statistically significant. These results were therefore considered exploratory and do not establish an allele dosage effect (**Supplementary Figure S5**). Overall, the LD analysis identified 65 pairs of HLA alleles associated with numerical and/or categorical differences following pp65 stimulation and 36 such pairs following CMV IE1 stimulation (**Figure 3**; **Supplementary Figure S4**; **Supplementary Tables S6**, **S7**, **S10**). The identification of alleles in LD that are also associated with antigen-induced responses highlights the importance of a haplotypic context; however, it does not establish which allele directly contributes to antigen presentation or T-cell activation. Several associated alleles may therefore reflect the same underlying genetic signal rather than independent effects. These findings should be interpreted as donor registry-level HLA background associations that may inform future donor prioritization studies, but require independent epitope-level validation to define causal peptide–HLA relationships. Despite this limitation, such findings provide valuable insights for donor selection strategies, particularly given the remarkable diversity of HLA alleles within the population.

### 3.5 Impact of HLA allelic variability on the EBV (consensus)-specific T-cell response

We analyzed the association between HLA genotype and T-cell response to a GMP-grade EBV consensus peptide pool composed of preselected immunodominant peptides derived from EBV proteins and annotated for defined HLA restrictions (HLA-A*02, A*03, A*11, A*24, A*26, B*07, B*08, B*15, B*18, B*27, B*35, B*40, B*44:02, DRB1*11:01, DR7, DR52a,b,c, DR15, DQ6, DR51, DP2, DP5, and DR14). These peptides, available in GMP-grade form, are currently used in the generation of immunotherapy products (16). Unlike the CMV pp65 and IE1 peptide pools, which consist of overlapping peptides covering full viral proteins, the EBV consensus pool contains selected immunodominant peptides with annotated HLA restrictions. Therefore, the EBV responses in this study should be interpreted as responses to this specific clinically relevant consensus peptide pool rather than as a comprehensive assessment of the total EBV-specific T-cell immunity.

Analysis of the T-cell response to EBV consensus showed that, among the HLA types evaluated, A*11:01, B*40:01, and B*44:02 were associated with a significantly higher T-cell response to this peptide pool (**Figure 4A**). Notably, these alleles are in strong LD with additional HLA variants also showing significant associations, such as HLA-C*05:01, C*03:04, DRB1*04:04, DPB1*03:01P, DPB1*06:01P, DQB1*03:02, and DQA1*03:01P (**Figure 4A**; **Supplementary Table S11**). Because of the strong LD structure of the HLA region, these associations cannot be assigned to a single causal allele and should be interpreted as linked HLA background associations. Despite peptides within this pool being previously reported as HLA-A*26:01 restricted, our results demonstrated categorically and numerically lower T-cell response for donors carrying this allele (**Figure 4A**; **Supplementary Figure S3**; **Supplementary Table S8**). This finding indicates that the presence of an annotated restricting allele does not necessarily predict a strong functional response in this assay, likely reflecting differences in epitope hierarchy, precursor frequency, T-cell receptor (TCR) repertoire, antigen exposure history, or the contribution of additional linked HLA alleles. Except for five individuals, all of the donors included in the analysis carried at least one HLA allele defined in the EBV consensus, confirming that the peptide pool was designed to achieve broad population coverage. Nevertheless, these five donors still exhibited moderate to high T-cell responses upon stimulation with the peptide pool (**Figure 4B**). This observation may reflect peptide recognition through additional HLA molecules not annotated for the pool, HLA peptide promiscuity, cross-reactive memory responses, or limitations in assigning restriction based only on manufacturer-annotated epitopes. Conversely, the possibility that EBV-specific responses are underestimated in donors whose dominant epitopes or restricting HLA molecules are not represented in the consensus pool cannot be excluded.

### 3.6 HLA-G 3′UTR variants and haplotypes associated with CMV-specific T-cell response

**Figure 5:**
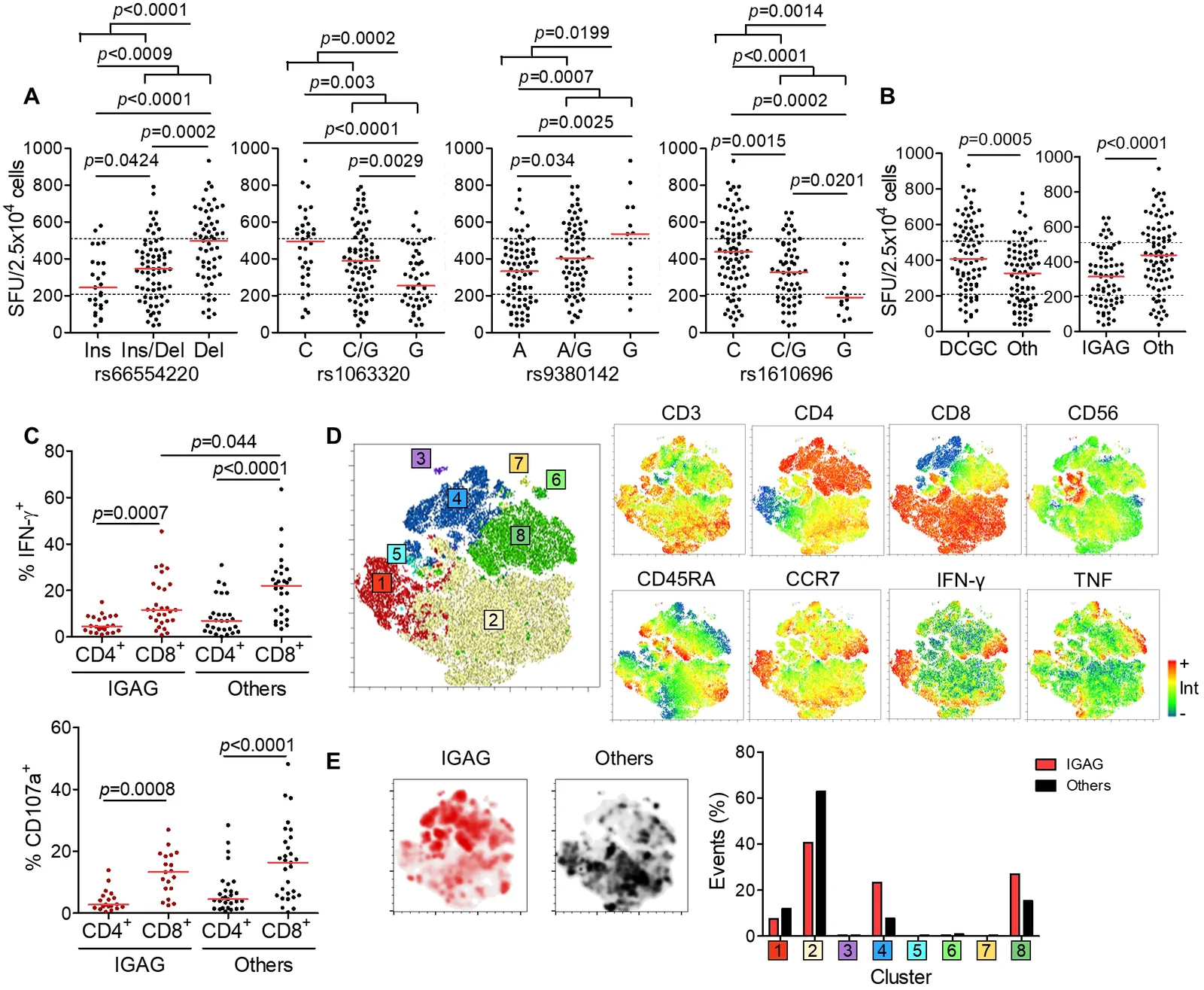
Cytomegalovirus (CMV)-specific T-cell response distributed by HLA-G 3′UTR variants and haplotypes. **(A, B)** IFN-γ spot forming unit (SFU) of 2.5 × 10^4^ cells stimulated with CMV pp65 distributed by four HLA-G 3′UTR variants **(A)** and by HLA-G 3′UTR haplotypes **(B)** (Mann–Whitney test or *t*-test). **(C)** Upper: Percentages of IFN-γ^+^ cells. Lower: CD107a^+^ cells among CD3^+^CD4^+^ or CD3^+^CD8^+^ cells T cells of the peripheral blood mononuclear cells (PBMCs) stimulated with the CMV pp65 peptide pool stratified by HLA-G 3′UTR haplotype (Mann–Whitney test or Wilcoxon matched-pairs test). **(D)** Left: t-Distributed stochastic neighbor embedding (t-SNE) unsupervised clustering with eight major clusters identified, numbered from *1* to *8*. Right: t-SNE colored by intensity signal, from high intensity in *red*, intermediate intensity in *green*, and low intensity in *blue*, of the flow cytometry data of CD3, CD4, CD8, CD56, CD45RA, CCR7, IFN-γ, and TNF among IFN-γ^+^ and IFN-γ^+^TNF^+^ cells after CMV pp65 stimulation from 27 donors (10 IGAG and 17 others). **(E)** Left: Density plots of the flow cytometry data from donors with (in *red*) or without (in *black*) the IGAG HLA-G 3′UTR haplotype. Right: Percentage of events within the clusters shown in **(D)** from donors with (in *red*) or without (in *black*) the IGAG HLA-G 3′UTR haplotype.

Given that the variability in the HLA-G 3′UTR has been reported as a risk factor for CMV infection in immunocompromised patients (17, 18), we investigated the association between the HLA-G 3′UTR genotypes included in our sequencing analysis and the IFN-γ ELISpot response. We analyzed the variants rs66554220, rs1063320, rs9380142, and rs1610696 included in our sequence coverage. The ELISpot results revealed that donors carrying the 14-bp insertion [rs66554220; either homozygous (Ins/Ins) or heterozygous (Ins/Del)] exhibited a significantly lower IFN-γ response compared with donors with the deletion homozygous genotype (Del/Del) (**Figure 5A**; **Supplementary Figure S6A**). The presence of the C allele (C/C or C/G) in rs1063320 was associated with a significantly higher number of SFUs following CMV pp65 stimulation, with a similar trend observed upon CMV IE1 stimulation (**Figure 5A**; **Supplementary Figure S6A**). Similarly, donors carrying at least one G allele at rs9380142 showed an enhanced T-cell response to pp65 (**Figure 5A**). In contrast, the presence of a G allele at rs1610696 correlated with lower IFN-γ SFU values (**Figure 5A**; **Supplementary Figure S6A**). Haplotype analysis indicated that the DCGC haplotype (rs66554220: Del, Ins/Del; rs1063320: C, C/G; rs9380142: G, G/A; rs1610696: C, C/G) was associated with a stronger response to CMV antigens, whereas the IGAG haplotype (rs66554220: Ins, Ins/Del; rs1063320: G, G/C; rs9380142: A, A/G; rs1610696: G, G/C) correlated with a weaker response (**Figure 5B**; **Supplementary Figure S6B**). In contrast, stimulation with consensus EBV peptides revealed no significant association between the HLA-G 3′UTR variants and the T-cell response (**Supplementary Figures S6C, D**). Consistent with previous reports that HLA-G 3′UTR polymorphisms affect HLA-G expression (19) and may consequently influence CD8^+^CD3^+^ T-cell function (20, 21), flow cytometry analysis of a subset of samples stimulated with CMV pp65 demonstrated that donors carrying the IGAG haplotype had a significantly lower proportion of IFN-γ-producing CD8^+^CD3^+^ T cells, while no difference was observed in CD107a expression (**Figure 5C**). The absence of a detectable difference in CD107a expression suggests that the association was more evident for IFN-γ production than for degranulation in this cohort. However, CD107a mobilization is an indirect functional readout and does not establish that cytotoxic activity was fully preserved. The reduction in CD8^+^CD3^+^ T-cell response was further supported using dimensionality reduction flow cytometry. Unsupervised clustering of IFN-γ^+^ and IFN-γ^+^TNF^+^ cells from 27 healthy donors, i.e., 10 IGAG and 17 others, following antigen stimulation distinguished the major CD4^+^ (clusters 4 and 8) and CD8^+^ (clusters 1 and 2) T-cell populations (**Figure 5D**). Notably, donors with the IGAG haplotype displayed a reduced frequency of CD8^+^ T-cell clusters (clusters 1 and 2) (**Figures 5D, E**). In contrast, the HLA-G 3′UTR variants showed no detectable effect on the distribution in general memory T-cell populations (**Figure 5D**; **Supplementary Figure S6E**). IGAG carriers showed a lower proportion of events in the CD8^+^ T-cell clusters across different memory profiles, including cluster 1, which exhibited a more central memory-like phenotype characterized by higher CCR7 expression. Overall, our results indicate that the HLA-G 3′UTR variants and haplotypes are associated with differences in CMV pp65-specific IFN-γ production by CD8^+^ T cells in healthy donors. These findings support a potential contribution of non-classical HLA variation to inter-donor variability in CMV-specific cellular immunity while requiring further mechanistic validation and confirmation in a larger independent cohort.

## 4 Discussion

CMV and EBV reactivation is a frequent infectious complication in immunocompromised patients (22). VST therapy represents an effective therapeutic strategy when antiviral pharmacotherapy is insufficient or contraindicated, initially in the setting of allogeneic hematopoietic stem cell transplantation and, increasingly, in solid organ transplantation, in individuals with primary or acquired immunodeficiencies, and in those with hematologic malignancies (23–26). In this context, optimal VST generation depends on the identification of healthy donors with both suitable HLA compatibility and robust virus-specific memory T-cell responses. Our study shows that classical HLA alleles, linked HLA haplotypic backgrounds, and non-classical HLA-G 3′UTR variations are associated with the magnitude and quality of CMV- and EBV-specific T-cell responses, supporting the integration of immunogenetic and functional data into donor selection strategies. Importantly, because donors were HLA-heterozygous and stimulated with multi-epitope peptide pools, the current study identifies donor registry-level associations rather than independent causal effects of individual HLA molecules.

The pronounced CD8^+^ T-cell bias observed after stimulation with the CMV pp65 and EBV consensus peptides highlights the relevance of cytotoxic T-cell responses against latent herpesvirus antigens, even when the peptide pools include both class I- and class II-restricted epitopes. This skew toward CD8^+^ activation for EBV peptides, despite their inclusive design, may reflect superior class I processing efficiency or epitope hierarchy favoring cytotoxic effectors in healthy donors. However, interpretation of the EBV responses should also consider that the EBV consensus pool is composed of selected immunodominant peptides with annotated HLA restrictions and does not represent the full EBV proteome. In contrast, CMV pp65 induced both CD4^+^ and CD8^+^ T-cell responses, consistent with its established role as a broadly immunogenic antigen containing epitopes recognized by both helper and cytotoxic T-cell compartments (8, 9, 27). The enrichment of T_EM_-like across responding populations further supports their relevance for rapid antiviral recall responses and VST manufacturing. Moreover, the correlation between the IFN-γ^+^ T_EM_-like frequencies measured by flow cytometry and the IFN-γ and ELISpot outputs supports the use of ELISpot as a scalable functional screening assay for donor evaluation. Inclusion of the CMV IE1 peptide pool revealed lower IFN-γ responses than pp65, together with only a weak correlation between the pp65- and IE1-specific responses. These findings reinforce pp65 as an immunodominant CMV target while supporting the complementary GMP-grade utility of IE1 in multi-antigen VST products (6, 28).

The analysis of serological status further showed that the CMV-specific IgG levels were higher in responders than in non-responders, but did not fully predict the magnitude of the cellular response. Some seropositive donors showed weak or absent pp65- or IE1-specific IFN-γ responses, whereas a small number of seronegative donors displayed low but detectable cellular responses. These low-level responses may reflect cross-reactive T cells generated by non-CMV antigenic exposures, previous CMV exposure not detected by serology, or assay-related sensitivity limitations (29). These discrepancies indicate that humoral and cellular antiviral immunity are not completely aligned and may be influenced by antigen exposure history, time since infection, subclinical reactivation, assay sensitivity, and the specific viral antigen analyzed (30, 31). This discordance between serology and cellular immunity has relevant clinical implications. In hematopoietic stem cell and solid organ transplantation, CMV risk stratification is commonly based on donor and recipient serostatus, which remains essential for defining exposure history and estimating the risk of primary infection or reactivation. However, the weak correlations observed in our cohort between the serological and cellular responses indicate that antibody levels explain only a limited proportion of the inter-donor variability in CMV-specific IFN-γ responses. Serology therefore provides useful information on exposure history, but should not be considered a quantitative surrogate for the magnitude, quality, or persistence of CMV-specific cellular immunity (32, 33). Functional assays such as IFN-γ ELISpot or related cellular immune monitoring platforms may therefore complement serology by identifying individuals with limited antiviral T-cell immunity despite previous exposure or by detecting low-level cellular reactivity not fully captured by antibody-based testing. In the context of VST therapy, combining serological and functional cellular data may support the identification of donors with more robust functional antiviral responses, whereas in transplantation it may refine recipient immune monitoring and risk stratification beyond conventional serological classification. VST production depends on the availability of suitable HLA-matched donors (34–37). Identifying donors with HLA alleles that efficiently present immunodominant epitopes can improve donor prioritization and product potency (6, 10). In our cohort, the associations of HLA-A*02:01 with the pp65-specific response and HLA-A*24:02 with the IE1-specific response are consistent with previous epitope studies (38, 39). In contrast to some previously reported population frequency–response relationships (8, 9), several common HLA-A and HLA-B alleles, including A*01:01 and B*07:01, were associated with reduced pp65-specific activation in our cohort. These findings should be interpreted cautiously, particularly for associations that did not remain significant after correction for multiple comparisons, and should be considered exploratory until validated in independent cohorts. Inter-donor variability may also reflect additional biological factors, including the timing of primary CMV infection, frequency of subclinical reactivation, antigen burden, viral load history, age, and time since last antigen exposure, all of which can shape the magnitude, phenotype, and functional state of CMV-specific memory T-cell responses (27, 30, 40, 41).

Previous studies have established HLA-A*02:01 and HLA-B*07:02 as major restricting molecules for immunodominant CMV pp65 epitopes (8, 42). However, the majority of these studies evaluated defined peptide–HLA combinations, tetramer-positive cells, or single HLA-restricted responses, whereas our study assessed the global IFN-γ response to an overlapping pp65 peptide pool in HLA-heterozygous donors. This distinction is relevant because, in a physiological donor setting, multiple HLA molecules may simultaneously present overlapping or distinct viral epitopes, generating a hierarchy of immunodominant responses rather than independent additive effects. Thus, the observation that donors carrying both HLA-A*02:01 and HLA-B*07:02 showed lower pp65-induced IFN-γ responses than A*02:01-only donors should be interpreted as evidence that the broader HLA background can modulate the response magnitude rather than as direct proof of molecular competition between these alleles.

One possible explanation is that the pp65-specific T-cell responses restricted by one allele may dominate the available memory repertoire, thereby limiting the expansion or detection of the responses restricted by another allele. Alternatively, linked HLA alleles or haplotypes, differences in precursor frequency, the TCR repertoire composition, the peptide-binding affinity, or the antigen exposure history may influence the final response measured by ELISpot. Confirmation would require epitope-level analyses using peptide–HLA tetramers, single-peptide stimulation, antigen-presenting cells expressing individual HLA molecules, or TCR repertoire tracking of allele-restricted clonotypes. Nevertheless, this finding is relevant for donor selection because it suggests that the presence of a known immunodominant HLA allele, such as HLA-A*02:01, may not be sufficient to predict the magnitude of the antiviral response without considering the complete HLA genotype and haplotypic context.

The low-response haplotype identified in this study further supports the importance of interpreting antiviral responses in the context of complete HLA backgrounds rather than isolated alleles. The common A*01:01~ C*07:01~ B*08:01~DRB1*03:01~DQB1*02:01 haplotype was associated with reduced pp65-specific responses, potentially reflecting combined effects of linked alleles, epitope hierarchy, or lower representation of strongly immunogenic pp65 epitopes within this HLA context. However, because this haplotype contains several linked alleles, the current analysis cannot determine whether the reduced response is attributable to one specific allele, the combined haplotypic background, or other linked genetic variants. This does not imply that A*01:01-positive donors are globally poor CMV responders as other CMV proteins, such as pp50, have been reported to induce strong responses in individuals carrying HLA-A*01:01 (43). This observation highlights the potential value of multi-antigen stimulation strategies for broadening antiviral coverage and avoiding the underestimation of donors whose responses are directed against non-pp65 antigens.

Several biological and methodological factors may also contribute to differences between our findings and those of previous reports. Firstly, the CMV-specific T-cell immunity is shaped by donor-specific variables that were not fully captured in this study, including the time since primary infection, the frequency of subclinical viral reactivation, viral load history, the inflammatory context, age, and the immunological status at the time of donation. Secondly, CMV seropositivity and IgG titers provide only partial information on cellular immunity and did not fully predict the magnitude of the ELISpot response in our cohort. Thirdly, our assay used thawed PBMCs stimulated for 6 days with overlapping peptide pools in the presence of IL-2, followed by IFN-γ ELISpot or intracellular cytokine staining. This approach is highly relevant for VST donor screening and manufacturing, but it differs from direct *ex vivo* tetramer staining, single-peptide stimulation, artificial antigen-presenting cell systems, or assays measuring cytotoxicity against infected targets. Finally, because peptide pools include multiple potential class I and class II epitopes, the measured response reflects the integrated outcome of antigen presentation, memory precursor frequency, proliferative capacity, and cytokine production. In addition, the large number of HLA comparisons increases the possibility of false-positive findings. For this reason, associations that did not remain significant after FDR correction should be interpreted as exploratory and hypothesis-generating. An additional consideration is the extensive LD across the HLA region. Because HLA alleles are inherited within extended haplotypes, the allele presence comparisons were not independent, and several associated alleles may tag the same underlying haplotypic signal. Although FDR correction reduced the risk of false-positive findings, it cannot distinguish the allele directly responsible for an association within an LD block or fully resolve the correlation among HLA tests. Therefore, associations involving linked alleles should be interpreted as HLA background signals rather than independent molecular effects.

For EBV, the consensus peptide pool induced broad reactivity across the donor cohort, supporting its relevance for VST manufacturing (44, 45). Among the HLA alleles evaluated, A*11:01, B*40:01, and B*44:02 were associated with stronger EBV-specific responses, together with additional variants in strong LD. These results suggest that specific HLA backgrounds may favor stronger responses to the EBV consensus peptide pool and could be informative for donor prioritization. Conversely, the reduced response observed among HLA-A*26:01 carriers, despite the inclusion of A*26-restricted peptides in the pool, suggests that the presence of a listed restricting allele does not necessarily guarantee a strong functional response. The observation that some donors lacking the annotated HLA restrictions still mounted detectable responses also suggests the presence of additional HLA–peptide interactions, allele promiscuity, or cross-presentation mechanisms (46). Therefore, our EBV data should not be interpreted as a comprehensive evaluation of global EBV-specific immunity but rather as an assessment of functional responses to a clinically relevant consensus peptide preparation. Overall, although the CMV and EBV responses showed a modest correlation, the HLA determinants driving memory T-cell formation and response magnitude appear to be at least partly virus- and antigen-specific.

From a practical donor screening perspective, our findings suggest a hypothesis-generating framework that could be evaluated in future donor prioritization studies for adoptive VST therapy. The first level is the intrinsic antiviral competence of the donor, reflected by the magnitude and quality of the CMV- or EBV-specific T-cell response after antigen stimulation. The second level is the functional applicability of that response in the recipient, which depends on whether the relevant restricting HLA allele is shared between donor and recipient. Thus, a donor carrying an immunodominant allele such as HLA-A*02:01 and displaying a strong pp65-specific response may not necessarily represent the optimal therapeutic option if the antiviral response is restricted by an HLA molecule absent in the recipient. Conversely, a donor with an intermediate response may be clinically preferable if the response is restricted through an HLA allele shared with the recipient and therefore capable of recognizing infected recipient cells. The present study did not prospectively compare HLA-guided and conventional donor selection strategies or assess whether the proposed approach improves VST manufacturing success, product functionality, persistence, antiviral activity, or clinical outcomes. Its clinical utility therefore remains to be established in prospective manufacturing and clinical studies. Nevertheless, future donor selection algorithms should therefore integrate HLA matching, known or predicted HLA restriction, response magnitude, antigen specificity, and product availability. In this context, our data provide a framework to refine VST bank annotation by combining donor HLA genotype with functional antiviral screening rather than ranking donors exclusively by either HLA type or ELISpot magnitude.

HLA-G is a non-classical HLA molecule with immunomodulatory properties (47). Variation in the HLA-G 3′UTR can influence transcriptional and posttranscriptional regulation, thereby affecting HLA-G expression and soluble levels of HLA-G (48, 49). Although HLA-G expression was not directly assessed, our findings extend previous evidence linking HLA-G 3′UTR polymorphisms with CMV infection outcomes in immunocompromised patients (17, 18) by showing that these variants are also associated with CMV-specific T-cell responses in healthy donors. Although in a small sample size, the DCGC haplotype was associated with stronger IFN-γ responses to CMV pp65, whereas IGAG was correlated with weaker responses and a reduced frequency of IFN-γ-producing CD8^+^ T cells. The lower frequency of IFN-γ-producing CD8^+^ T cells in IGAG carriers was not accompanied by a detectable difference in CD107a expression, suggesting a more selective association with cytokine production than with degranulation because CD107a is a surrogate marker of granule exocytosis rather than a direct measure of target cell killing (50). Given the limited cohort size, these observations should therefore be considered preliminary and require confirmation using larger cohorts and direct cytotoxicity assays. These haplotypes correspond to two major HLA-G 3′UTR, UTR-1 and UTR-2 (51). Notably, LD between HLA-G 3′UTR haplotypes and HLA-G alleles may underlie these differences. UTR-2, associated with HLA-G*01:06 (52), an allele in our cohort with a lower CMV pp65-specific T-cell response, may suggest a possible contribution of linked HLA-G variation to the observed response pattern. However, the present study cannot determine whether this association reflects a direct functional effect of HLA-G*01:06, the HLA-G 3′UTR haplotype, or other linked genetic variants. These effects were specific to CMV, but absent after EBV stimulation, indicating virus-specific interaction between HLA-G polymorphisms and T-cell immunity. In translational terms, HLA-G 3′UTR variability should be interpreted as a complementary biomarker rather than as a stand-alone donor selection criterion. Classical HLA compatibility, shared restrictive HLA alleles, antigen specificity, response magnitude, and the capacity of the infused cells to expand and persist *in vivo* are likely to remain the dominant determinants of clinical efficacy. Nevertheless, the association between HLA-G 3′UTR haplotypes and CMV-specific IFN-γ production suggests that non-classical HLA variation may contribute to inter-donor differences in antiviral T-cell responsiveness. Therefore, HLA-G genotyping could potentially be incorporated as an additional variable in future donor prioritization models, particularly when several otherwise comparable donors are available. In such a model, the HLA-G status would not replace classical HLA matching or functional antiviral testing, but could help refine donor ranking by identifying genetic backgrounds associated with stronger or weaker CMV-specific cellular responses.

The generalizability of these findings should be considered in the context of population-specific HLA diversity. The donor cohort analyzed here showed HLA allele and haplotype frequencies comparable to those reported in the Spanish population, supporting the relevance of our observations for local donor registry optimization. However, the HLA allele frequencies, LD patterns, and immunodominant haplotypes vary substantially across geographical regions and ethnic groups (53). Therefore, while the central conclusion that classical and non-classical HLA variations are associated with antiviral T-cell responsiveness is likely broadly applicable, the specific alleles or haplotypes associated with stronger or weaker responses may differ in other populations. Replication in independent and ethnically diverse cohorts will therefore be required before these findings can be incorporated into broadly applicable donor selection algorithms. Consequently, our results should be viewed as a population-informed framework rather than a universal allele ranking system. Replication in ethnically diverse cohorts and integration with local HLA registry data will be necessary to adapt these findings to different clinical settings and to maximize the coverage of third-party VST banks.

This study has several limitations. First, it was performed in healthy donors and measured *in vitro* IFN-γ responses; therefore, it cannot directly establish whether high-response HLA genotypes translate into improved viral clearance, reduced viral recurrence, or better survival in patients receiving VST therapy. Clinical efficacy is dependent on multiple additional variables, including donor–recipient HLA compatibility, the restricting HLA allele of the infused T cells, antigen specificity, functional avidity, cell dose, *in vivo* persistence, recipient immunosuppression, baseline lymphocyte counts, and disease burden (3, 54). Second, although the use of overlapping peptide pools is relevant for VST donor screening, it does not define the exact peptide–HLA restriction of each response. Third, the high polymorphism and the LD structure of the HLA region limit causal assignment to individual alleles, as several associated alleles or variants may represent the same underlying haplotypic signal. Fourth, because donors were HLA-heterozygous and each individual carried multiple HLA class I and class II alleles, the presence-*versus*-absence comparisons used in this study cannot isolate the independent contribution of a single HLA molecule. Ideally, this would require comparison of donors differing in only one HLA allele while sharing all other HLA alleles, which is not feasible in a cohort of this size given the diversity and the LD structure of the HLA region. Fifth, the large number of HLA comparisons increases the risk of false-positive findings. We therefore applied FDR correction and interpret nominal associations that did not survive correction as exploratory. Although multiple testing correction reduces the risk of false-positive findings, it cannot distinguish causal alleles from correlated markers within the same LD block. Sixth, the EBV consensus peptide pool contains selected immunodominant peptides with annotated HLA restrictions rather than full-protein coverage and may therefore underestimate the EBV-specific responses in donors whose dominant epitopes or restricting HLA molecules are not represented. Seventh, CCR7/CD45RA phenotyping was performed after antigen-driven culture and re-stimulation and therefore reflects culture-associated differentiation phenotypes rather than definitive *ex vivo* memory states. Thus, although our data provide a rational basis for the incorporation of donor HLA genotype and antigen-specific functional screening into VST bank design, the reported associations require validation in independent populations and through haplotype- and epitope-level analyses, and prospective clinical validation will be required to determine how these variables should be weighted in donor selection algorithms.

In conclusion, this study identifies classical and non-classical HLA genetic features associated with CMV- and EBV-specific T-cell responses in healthy donors. HLA alleles, linked haplotypic background, and HLA-G 3′UTR polymorphisms were associated with antiviral T-cell activation in an antigen- and virus-dependent manner, emphasizing the multifactorial nature of donor immune competence. These findings support the integration of HLA genotype, functional antiviral screening, and population-specific donor registry data to optimize VST donor selection, peptide pool design, and prediction of antiviral potential in research and clinical settings.

## Data Availability

The original contributions presented in the study are included in the article/**Supplementary Material**. Further inquiries can be directed to the corresponding authors.
