# Supplementary material for: Human leukocyte antigen genotype modulates cytomegalovirus- and Epstein–Barr virus-specific T-cell response in healthy donors for optimized virus-specific T-cell donor selection
Source: Front Immunol. 2026 Aug 14;17:1856550. doi: 10.3389/fimmu.2026.1856550 (PMC13539606; doi:10.3389/fimmu.2026.1856550)
Supplement: Supplementary file 1 [file SupplementaryFile1.docx]

**Table S1.** Characteristics of the donors included in the flow cytometry analysis. The table includes sex, age, serological status, and IFN-γ ELISpot response; non-responders (NR), low (LR), medium (MR), or high (HR) responders. Minimum, maximum, and median (m) values are shown.

|  | Donors | Gender  F/M | Age  Years | CMV  IgG^+^ | EBV  IgG^+^ | ELISpot  NR/LR/MR/HR |
| --- | --- | --- | --- | --- | --- | --- |
| CMV pp65 | 57 | 34/23 | 21-55 (m=29) | 51  (89.5%) | 56  (98.2%) | 6/18/18/15 |
| EBV | 51 | 32/19 | 21-55  (m=30) | 43  (84.3%) | 50  (98%) | 2/9/26/14 |

**Table S2.** Frequencies of HLA alleles in the donors included in the study. Allele Frequency (AF).

| HLA-A | AF | HLA-B | AF | HLA-C | AF |
| --- | --- | --- | --- | --- | --- |
| *A*02:01* | 0.2450 | *B*08:01* | 0.1188 | *C*07:01* | 0.1832 |
| *A*01:01* | 0.1312 | *B*44:03* | 0.1015 | *C*05:01* | 0.1015 |
| *A*24:02* | 0.1287 | *B*18:01* | 0.0891 | *C*04:01* | 0.0990 |
| *A*03:01* | 0.0817 | *B*07:02* | 0.0817 | *C*07:02* | 0.0916 |
| *A*29:02* | 0.0817 | *B*51:01* | 0.0594 | *C*16:01* | 0.0891 |
| *A*11:01* | 0.0619 | *B*44:02* | 0.0446 | *C*06:02* | 0.0817 |
| *A*30:02* | 0.0495 | *B*14:02* | 0.0421 | *C*12:03* | 0.0693 |
| *A*26:01* | 0.0322 | *B*49:01* | 0.0371 | *C*08:02* | 0.0520 |
| *A*23:01* | 0.0297 | *B*57:01* | 0.0347 | *C*02:02* | 0.0446 |
| *A*32:01* | 0.0297 | *B*38:01* | 0.0347 | *C*03:04* | 0.0272 |
| *A*31:01* | 0.0173 | *B*35:01* | 0.0322 | *C*01:02* | 0.0248 |
| *A*02:05* | 0.0173 | *B*15:01* | 0.0272 | *C*15:02* | 0.0223 |
| *A*68:01* | 0.0149 | *B*40:01* | 0.0272 | *C*03:03* | 0.0198 |
| *A*33:01* | 0.0124 | *B*35:03* | 0.0198 | *C*15:05* | 0.0149 |
| *A*68:02* | 0.0099 | *B*13:02* | 0.0198 | *C*07:18* | 0.0149 |
| *A*25:01* | 0.0099 | *B*40:02* | 0.0198 | *C*12:02* | 0.0124 |
| *A*29:01* | 0.0074 | *B*27:05* | 0.0198 | *C*07:04* | 0.0074 |
| *A*69:01* | 0.0074 | *B*14:01* | 0.0173 | *C*16:02* | 0.0074 |
| *A*34:02* | 0.0050 | *B*58:01* | 0.0173 | *C*14:02* | 0.0050 |
| *A*01:02* | 0.0050 | *B*45:01* | 0.0149 | *C*17:03* | 0.0050 |
| *A*30:04* | 0.0050 | *B*52:01* | 0.0149 | *C*17:01* | 0.0050 |
| *A*02:02* | 0.0050 | *B*37:01* | 0.0149 | *C*03:05* | 0.0046 |
| *A*80:01* | 0.0025 | *B*39:01* | 0.0124 | *C*03:02* | 0.0025 |
| *A*29:10* | 0.0025 | *B*50:01* | 0.0124 | *C*15:13* | 0.0025 |
| *A*02:06* | 0.0025 | *B*07:05* | 0.0074 | *C*03:07* | 0.0025 |
| *A*30:01* | 0.0025 | *B*55:01* | 0.0074 | *C*15:04* | 0.0025 |
| *A*24:03* | 0.0025 | *B*35:08* | 0.0074 | *C*04:03* | 0.0025 |
|  |  | *B*67:01* | 0.0050 | *C*16:04* | 0.0025 |
|  |  | *B*47:01* | 0.0050 | *C*08:04* | 0.0025 |
|  |  | *B*35:02* | 0.0050 |  |  |
|  |  | *B*15:17* | 0.0050 |  |  |
|  |  | *B*41:02* | 0.0050 |  |  |
|  |  | *B*15:18* | 0.0050 |  |  |
|  |  | *B*41:01* | 0.0050 |  |  |
|  |  | *B*56:01* | 0.0025 |  |  |
|  |  | *B*53:01* | 0.0025 |  |  |
|  |  | *B*51:05* | 0.0025 |  |  |
|  |  | *B*57:03* | 0.0025 |  |  |
|  |  | *B*39:06* | 0.0025 |  |  |
|  |  | *B*44:05* | 0.0025 |  |  |
|  |  | *B*44:302* | 0.0025 |  |  |
|  |  | *B*27:07* | 0.0025 |  |  |
|  |  | *B*07:06* | 0.0025 |  |  |
|  |  | *B*35:43* | 0.0025 |  |  |
|  |  | *B*13:01* | 0.0025 |  |  |
|  |  | *B*15:10* | 0.0025 |  |  |

| HLA-DRB1 | AF | HLA-DQA1 | AF | HLA-DQB1 | AF | HLA-DPA1 | AF | HLA-DPB1 | AF |
| --- | --- | --- | --- | --- | --- | --- | --- | --- | --- |
| *DRB1*03:01* | 0.1990 | *DQA1*05:01P* | 0.3187 | *DQB1*02:01* | 0.1955 | *DPA1*01:03P* | 0.7345 | *DPB1*04:01P* | 0.3342 |
| *DRB1*07:01* | 0.1816 | *DQA1*01:01P* | 0.1896 | *DQB1*05:01* | 0.1634 | *DPA1*02:01P* | 0.2401 | *DPB1*02:01P* | 0.1390 |
| *DRB1*01:01* | 0.0896 | *DQA1*02:01P* | 0.1621 | *DQB1*02:02* | 0.1584 | *DPA1*02:02P* | 0.0254 | *DPB1*03:01P* | 0.0963 |
| *DRB1*15:01* | 0.0821 | *DQA1*01:02P* | 0.1484 | *DQB1*03:01* | 0.1213 |  |  | *DPB1*04:02P* | 0.0909 |
| *DRB1*13:01* | 0.0473 | *DQA1*03:01P* | 0.1016 | *DQB1*06:02* | 0.0792 |  |  | *DPB1*01:01P* | 0.0775 |
| *DRB1*13:02* | 0.0423 | *DQA1*04:01P* | 0.0247 | *DQB1*03:02* | 0.0718 |  |  | *DPB1*11:01P* | 0.0481 |
| *DRB1*01:02* | 0.0398 |  |  | *DQB1*06:03* | 0.0495 |  |  | *DPB1*02:02P* | 0.0401 |
| *DRB1*11:01* | 0.0398 |  |  | *DQB1*06:04* | 0.0322 |  |  | *DPB1*17:01P* | 0.0374 |
| *DRB1*11:04* | 0.0299 |  |  | *DQB1*04:02* | 0.0272 |  |  | *DPB1*10:01P* | 0.0267 |
| *DRB1*04:02* | 0.0249 |  |  | *DQB1*03:03* | 0.0248 |  |  | *DPB1*13:01P* | 0.0267 |
| *DRB1*04:04* | 0.0249 |  |  | *DQB1*05:02* | 0.0223 |  |  | *DPB1*14:01P* | 0.0214 |
| *DRB1*08:01* | 0.0224 |  |  | *DQB1*05:03* | 0.0149 |  |  | *DPB1*05:01P* | 0.0187 |
| *DRB1*16:01* | 0.0199 |  |  | *DQB1*06:01* | 0.0124 |  |  | *DPB1*06:01P* | 0.0160 |
| *DRB1*14:54* | 0.0174 |  |  | *DQB1*06:09* | 0.0124 |  |  | *DPB1*20:01P* | 0.0053 |
| *DRB1*12:01* | 0.0174 |  |  | *DQB1*03:19* | 0.0124 |  |  | *DPB1*09:01P* | 0.0053 |
| *DRB1*10:01* | 0.0149 |  |  | *DQB1*03:289* | 0.0025 |  |  | *DPB1*15:01P* | 0.0053 |
| *DRB1*04:01* | 0.0149 |  |  |  |  |  |  | *DPB1*19:01P* | 0.0027 |
| *DRB1*11:02* | 0.0124 |  |  |  |  |  |  | *DPB1*16:01P* | 0.0027 |
| *DRB1*04:05* | 0.0100 |  |  |  |  |  |  | *DPB1*39:01P* | 0.0027 |
| *DRB1*15:02* | 0.0100 |  |  |  |  |  |  | *DPB1*46:01P* | 0.0027 |
| *DRB1*13:03* | 0.0075 |  |  |  |  |  |  |  |  |
| *DRB1*01:03* | 0.0075 |  |  |  |  |  |  |  |  |
| *DRB1*04:07* | 0.0075 |  |  |  |  |  |  |  |  |
| *DRB1*04:03* | 0.0075 |  |  |  |  |  |  |  |  |
| *DRB1*14:01* | 0.0050 |  |  |  |  |  |  |  |  |
| *DRB1*11:03* | 0.0050 |  |  |  |  |  |  |  |  |
| *DRB1*03:02* | 0.0025 |  |  |  |  |  |  |  |  |
| *DRB1*09:01* | 0.0025 |  |  |  |  |  |  |  |  |
| *DRB1*04:11* | 0.0025 |  |  |  |  |  |  |  |  |
| *DRB1*12:03* | 0.0025 |  |  |  |  |  |  |  |  |
| *DRB1*11:14* | 0.0025 |  |  |  |  |  |  |  |  |
| *DRB1*04:92* | 0.0025 |  |  |  |  |  |  |  |  |
| *DRB1*08:02* | 0.0025 |  |  |  |  |  |  |  |  |
| *DRB1*04:08* | 0.0025 |  |  |  |  |  |  |  |  |

| HLA-E | AF | HLA-G | AF |
| --- | --- | --- | --- |
| *E*01:01* | 0.5263 | *G*01:01* | 0.7283 |
| *E*01:03* | 0.4632 | *G*01:04* | 0.1440 |
| *E*01:06* | 0.0079 | *G*01:06* | 0.0625 |
| *E*01:09* | 0.0026 | *G*01:03* | 0.0598 |
|  |  | *G*01:05N* | 0.0027 |
|  |  | *G*01:08* | 0.0027 |

**Table S3.** Frequencies of HLA alleles in the donors stimulated with CMV pp65 peptide pool. Allele Frequency (AF).

| HLA-A | AF | HLA-B | AF | HLA-C | AF |
| --- | --- | --- | --- | --- | --- |
| *A*02:01* | 0.2470 | *B*08:01* | 0.1280 | *C*07:01* | 0.1890 |
| *A*01:01* | 0.1372 | *B*44:03* | 0.0976 | *C*07:02* | 0.1037 |
| *A*24:02* | 0.1311 | *B*07:02* | 0.0915 | *C*04:01* | 0.0976 |
| *A*29:02* | 0.0854 | *B*18:01* | 0.0762 | *C*16:01* | 0.0884 |
| *A*03:01* | 0.0732 | *B*51:01* | 0.0549 | *C*06:02* | 0.0854 |
| *A*11:01* | 0.0610 | *B*57:01* | 0.0366 | *C*05:01* | 0.0854 |
| *A*30:02* | 0.0427 | *B*49:01* | 0.0366 | *C*12:03* | 0.0610 |
| *A*26:01* | 0.0366 | *B*15:01* | 0.0335 | *C*02:02* | 0.0518 |
| *A*32:01* | 0.0305 | *B*14:02* | 0.0335 | *C*08:02* | 0.0427 |
| *A*23:01* | 0.0213 | *B*44:02* | 0.0335 | *C*03:04* | 0.0305 |
| *A*31:01* | 0.0213 | *B*35:01* | 0.0305 | *C*01:02* | 0.0244 |
| *A*68:01* | 0.0183 | *B*40:01* | 0.0305 | *C*03:03* | 0.0244 |
| *A*02:05* | 0.0183 | *B*38:01* | 0.0274 | *C*15:05* | 0.0183 |
| *A*25:01* | 0.0122 | *B*40:02* | 0.0244 | *C*07:18* | 0.0183 |
| *A*29:01* | 0.0091 | *B*35:03* | 0.0213 | *C*15:02* | 0.0183 |
| *A*68:02* | 0.0091 | *B*58:01* | 0.0213 | *C*12:02* | 0.0122 |
| *A*69:01* | 0.0091 | *B*14:01* | 0.0183 | *C*16:02* | 0.0091 |
| *A*34:02* | 0.0061 | *B*13:02* | 0.0183 | *C*07:04* | 0.0061 |
| *A*33:01* | 0.0061 | *B*27:05* | 0.0183 | *C*17:03* | 0.0061 |
| *A*30:04* | 0.0061 | *B*45:01* | 0.0152 | *C*03:05* | 0.0057 |
| *A*80:01* | 0.0030 | *B*39:01* | 0.0152 | *C*14:02* | 0.0030 |
| *A*01:02* | 0.0030 | *B*52:01* | 0.0152 | *C*03:02* | 0.0030 |
| *A*02:06* | 0.0030 | *B*37:01* | 0.0152 | *C*15:13* | 0.0030 |
| *A*30:01* | 0.0030 | *B*50:01* | 0.0122 | *C*03:07* | 0.0030 |
| *A*02:02* | 0.0030 | *B*07:05* | 0.0091 | *C*17:01* | 0.0030 |
| *A*24:03* | 0.0030 | *B*55:01* | 0.0091 | *C*15:04* | 0.0030 |
|  |  | *B*35:08* | 0.0091 | *C*04:03* | 0.0030 |
|  |  | *B*47:01* | 0.0061 |  |  |
|  |  | *B*35:02* | 0.0061 |  |  |
|  |  | *B*15:17* | 0.0061 |  |  |
|  |  | *B*41:02* | 0.0061 |  |  |
|  |  | *B*56:01* | 0.0030 |  |  |
|  |  | *B*67:01* | 0.0030 |  |  |
|  |  | *B*53:01* | 0.0030 |  |  |
|  |  | *B*51:05* | 0.0030 |  |  |
|  |  | *B*57:03* | 0.0030 |  |  |
|  |  | *B*39:06* | 0.0030 |  |  |
|  |  | *B*44:05* | 0.0030 |  |  |
|  |  | *B*44:302* | 0.0030 |  |  |
|  |  | *B*27:07* | 0.0030 |  |  |
|  |  | *B*07:06* | 0.0030 |  |  |
|  |  | *B*35:43* | 0.0030 |  |  |
|  |  | *B*41:01* | 0.0030 |  |  |
|  |  | *B*13:01* | 0.0030 |  |  |
|  |  | *B*15:18* | 0.0030 |  |  |

| HLA-DRB1 | AF | HLA-DQA1 | AF | HLA-DQB1 | AF | HLA-DPA1 | AF | HLA-DPB1 | AF |
| --- | --- | --- | --- | --- | --- | --- | --- | --- | --- |
| *DRB1*03:01* | 0.2025 | *DQA1*05:01P* | 0.3322 | *DQB1*02:01* | 0.1982 | *DPA1*01:03P* | 0.7345 | *DPB1*04:01P* | 0.3333 |
| *DRB1*07:01* | 0.1810 | *DQA1*01:01P* | 0.1711 | *DQB1*02:02* | 0.1555 | *DPA1*02:01P* | 0.2345 | *DPB1*02:01P* | 0.1307 |
| *DRB1*15:01* | 0.0920 | *DQA1*01:02P* | 0.1611 | *DQB1*05:01* | 0.1463 | *DPA1*02:02P* | 0.0310 | *DPB1*03:01P* | 0.1078 |
| *DRB1*01:01* | 0.0798 | *DQA1*02:01P* | 0.1577 | *DQB1*03:01* | 0.1220 |  |  | *DPB1*04:02P* | 0.1046 |
| *DRB1*13:02* | 0.0491 | *DQA1*03:01P* | 0.1040 | *DQB1*06:02* | 0.0884 |  |  | *DPB1*01:01P* | 0.0817 |
| *DRB1*13:01* | 0.0460 | *DQA1*04:01P* | 0.0235 | *DQB1*03:02* | 0.0762 |  |  | *DPB1*11:01P* | 0.0458 |
| *DRB1*11:01* | 0.0368 |  |  | *DQB1*06:03* | 0.0488 |  |  | *DPB1*02:02P* | 0.0327 |
| *DRB1*01:02* | 0.0337 |  |  | *DQB1*06:04* | 0.0335 |  |  | *DPB1*17:01P* | 0.0294 |
| *DRB1*11:04* | 0.0276 |  |  | *DQB1*03:03* | 0.0305 |  |  | *DPB1*10:01P* | 0.0261 |
| *DRB1*04:04* | 0.0276 |  |  | *DQB1*04:02* | 0.0244 |  |  | *DPB1*14:01P* | 0.0229 |
| *DRB1*04:02* | 0.0215 |  |  | *DQB1*05:02* | 0.0244 |  |  | *DPB1*13:01P* | 0.0229 |
| *DRB1*12:01* | 0.0215 |  |  | *DQB1*06:09* | 0.0152 |  |  | *DPB1*05:01P* | 0.0196 |
| *DRB1*16:01* | 0.0215 |  |  | *DQB1*06:01* | 0.0122 |  |  | *DPB1*06:01P* | 0.0131 |
| *DRB1*04:01* | 0.0184 |  |  | *DQB1*03:19* | 0.0122 |  |  | *DPB1*20:01P* | 0.0065 |
| *DRB1*08:01* | 0.0184 |  |  | *DQB1*05:03* | 0.0091 |  |  | *DPB1*09:01P* | 0.0065 |
| *DRB1*14:54* | 0.0153 |  |  | *DQB1*03:289* | 0.0030 |  |  | *DPB1*19:01P* | 0.0033 |
| *DRB1*04:05* | 0.0123 |  |  |  |  |  |  | *DPB1*16:01P* | 0.0033 |
| *DRB1*10:01* | 0.0123 |  |  |  |  |  |  | *DPB1*39:01P* | 0.0033 |
| *DRB1*11:02* | 0.0123 |  |  |  |  |  |  | *DPB1*15:01P* | 0.0033 |
| *DRB1*13:03* | 0.0092 |  |  |  |  |  |  | *DPB1*46:01P* | 0.0033 |
| *DRB1*15:02* | 0.0092 |  |  |  |  |  |  |  |  |
| *DRB1*01:03* | 0.0092 |  |  |  |  |  |  |  |  |
| *DRB1*04:03* | 0.0092 |  |  |  |  |  |  |  |  |
| *DRB1*04:07* | 0.0061 |  |  |  |  |  |  |  |  |
| *DRB1*11:03* | 0.0061 |  |  |  |  |  |  |  |  |
| *DRB1*03:02* | 0.0031 |  |  |  |  |  |  |  |  |
| *DRB1*09:01* | 0.0031 |  |  |  |  |  |  |  |  |
| *DRB1*14:01* | 0.0031 |  |  |  |  |  |  |  |  |
| *DRB1*04:11* | 0.0031 |  |  |  |  |  |  |  |  |
| *DRB1*11:14* | 0.0031 |  |  |  |  |  |  |  |  |
| *DRB1*04:92* | 0.0031 |  |  |  |  |  |  |  |  |
| *DRB1*08:02* | 0.0031 |  |  |  |  |  |  |  |  |

| HLA-E | AF | HLA-G | AF |
| --- | --- | --- | --- |
| *E*01:01* | 0.5325 | *G*01:01* | 0.7349 |
| *E*01:03* | 0.4578 | *G*01:04* | 0.1376 |
| *E*01:06* | 0.0065 | *G*01:06* | 0.0638 |
| *E*01:09* | 0.0032 | *G*01:03* | 0.0570 |
|  |  | *G*01:05N* | 0.0034 |
|  |  | *G*01:08* | 0.0034 |

**Table S4.** Frequencies of HLA alleles in the donors stimulated with CMV IE1 peptide pool. Allele Frequency (AF).

| HLA-A | AF | HLA-B | AF | HLA-C | AF |
| --- | --- | --- | --- | --- | --- |
| *A*02:01* | 0.2260 | *B*07:02* | 0.1346 | *C*07:01* | 0.1635 |
| *A*01:01* | 0.1394 | *B*08:01* | 0.1058 | *C*07:02* | 0.1490 |
| *A*24:02* | 0.1106 | *B*18:01* | 0.0913 | *C*05:01* | 0.1058 |
| *A*03:01* | 0.0817 | *B*44:03* | 0.0865 | *C*04:01* | 0.0962 |
| *A*29:02* | 0.0817 | *B*51:01* | 0.0433 | *C*06:02* | 0.0817 |
| *A*11:01* | 0.0769 | *B*40:01* | 0.0385 | *C*16:01* | 0.0721 |
| *A*30:02* | 0.0529 | *B*49:01* | 0.0385 | *C*12:03* | 0.0673 |
| *A*26:01* | 0.0337 | *B*44:02* | 0.0385 | *C*03:04* | 0.0385 |
| *A*32:01* | 0.0288 | *B*57:01* | 0.0288 | *C*08:02* | 0.0337 |
| *A*23:01* | 0.0240 | *B*15:01* | 0.0288 | *C*02:02* | 0.0337 |
| *A*02:05* | 0.0192 | *B*14:02* | 0.0288 | *C*01:02* | 0.0240 |
| *A*25:01* | 0.0192 | *B*35:01* | 0.0288 | *C*07:18* | 0.0240 |
| *A*31:01* | 0.0144 | *B*38:01* | 0.0288 | *C*03:03* | 0.0240 |
| *A*69:01* | 0.0144 | *B*58:01* | 0.0288 | *C*15:02* | 0.0192 |
| *A*68:01* | 0.0096 | *B*27:05* | 0.0240 | *C*15:05* | 0.0144 |
| *A*68:02* | 0.0096 | *B*39:01* | 0.0240 | *C*12:02* | 0.0096 |
| *A*30:04* | 0.0096 | *B*37:01* | 0.0192 | *C*17:01* | 0.0096 |
| *A*02:02* | 0.0096 | *B*52:01* | 0.0144 | *C*14:02* | 0.0048 |
| *A*80:01* | 0.0048 | *B*35:03* | 0.0144 | *C*07:04* | 0.0048 |
| *A*29:01* | 0.0048 | *B*35:08* | 0.0144 | *C*03:02* | 0.0048 |
| *A*01:02* | 0.0048 | *B*13:02* | 0.0144 | *C*16:02* | 0.0048 |
| *A*02:06* | 0.0048 | *B*07:05* | 0.0096 | *C*17:03* | 0.0048 |
| *A*33:01* | 0.0048 | *B*47:01* | 0.0096 | *C*15:13* | 0.0048 |
| *A*30:01* | 0.0048 | *B*14:01* | 0.0096 | *C*15:04* | 0.0048 |
| *A*34:02* | 0.0048 | *B*55:01* | 0.0096 |  |  |
| *A*24:03* | 0.0048 | *B*35:02* | 0.0096 |  |  |
|  |  | *B*40:02* | 0.0096 |  |  |
|  |  | *B*41:01* | 0.0096 |  |  |
|  |  | *B*50:01* | 0.0096 |  |  |
|  |  | *B*67:01* | 0.0048 |  |  |
|  |  | *B*53:01* | 0.0048 |  |  |
|  |  | *B*57:03* | 0.0048 |  |  |
|  |  | *B*39:06* | 0.0048 |  |  |
|  |  | *B*44:05* | 0.0048 |  |  |
|  |  | *B*27:07* | 0.0048 |  |  |
|  |  | *B*44:302* | 0.0048 |  |  |
|  |  | *B*41:02* | 0.0048 |  |  |
|  |  | *B*15:17* | 0.0048 |  |  |
|  |  | *B*45:01* | 0.0048 |  |  |

| HLA-DRB1 | AF | HLA-DQA1 | AF | HLA-DQB1 | AF | HLA-DPA1 | AF | HLA-DPB1 | AF |
| --- | --- | --- | --- | --- | --- | --- | --- | --- | --- |
| *DRB1*03:01* | 0.1971 | *DQA1*05:01P* | 0.3385 | *DQB1*02:01* | 0.1971 | *DPA1*01:03P* | 0.7143 | *DPB1*04:01P* | 0.3367 |
| *DRB1*07:01* | 0.1442 | *DQA1*01:02P* | 0.1927 | *DQB1*05:01* | 0.1346 | *DPA1*02:01P* | 0.2582 | *DPB1*02:01P* | 0.1480 |
| *DRB1*15:01* | 0.1202 | *DQA1*01:01P* | 0.1458 | *DQB1*03:01* | 0.1346 | *DPA1*02:02P* | 0.0275 | *DPB1*03:01P* | 0.1020 |
| *DRB1*01:01* | 0.0673 | *DQA1*02:01P* | 0.1354 | *DQB1*02:02* | 0.1346 |  |  | *DPB1*04:02P* | 0.0765 |
| *DRB1*13:02* | 0.0481 | *DQA1*03:01P* | 0.1198 | *DQB1*06:02* | 0.1202 |  |  | *DPB1*01:01P* | 0.0714 |
| *DRB1*13:01* | 0.0433 | *DQA1*01:03P* | 0.0521 | *DQB1*03:02* | 0.0913 |  |  | *DPB1*17:01P* | 0.0459 |
| *DRB1*11:04* | 0.0385 | *DQA1*04:01P* | 0.0156 | *DQB1*06:03* | 0.0385 |  |  | *DPB1*02:02P* | 0.0408 |
| *DRB1*04:04* | 0.0385 |  |  | *DQB1*06:04* | 0.0337 |  |  | *DPB1*11:01P* | 0.0357 |
| *DRB1*01:02* | 0.0288 |  |  | *DQB1*05:02* | 0.0240 |  |  | *DPB1*10:01P* | 0.0306 |
| *DRB1*12:01* | 0.0288 |  |  | *DQB1*03:03* | 0.0192 |  |  | *DPB1*05:01P* | 0.0255 |
| *DRB1*11:01* | 0.0288 |  |  | *DQB1*04:02* | 0.0192 |  |  | *DPB1*14:01P* | 0.0255 |
| *DRB1*04:02* | 0.0240 |  |  | *DQB1*06:09* | 0.0144 |  |  | *DPB1*13:01P* | 0.0255 |
| *DRB1*16:01* | 0.0240 |  |  | *DQB1*03:19* | 0.0144 |  |  | *DPB1*06:01P* | 0.0204 |
| *DRB1*04:05* | 0.0192 |  |  | *DQB1*05:03* | 0.0096 |  |  | *DPB1*09:01P* | 0.0102 |
| *DRB1*08:01* | 0.0192 |  |  | *DQB1*06:01* | 0.0096 |  |  | *DPB1*20:01P* | 0.0051 |
| *DRB1*10:01* | 0.0144 |  |  | *DQB1*03:289* | 0.0048 |  |  |  |  |
| *DRB1*14:54* | 0.0144 |  |  |  |  |  |  |  |  |
| *DRB1*04:01* | 0.0144 |  |  |  |  |  |  |  |  |
| *DRB1*01:03* | 0.0144 |  |  |  |  |  |  |  |  |
| *DRB1*04:03* | 0.0144 |  |  |  |  |  |  |  |  |
| *DRB1*11:02* | 0.0144 |  |  |  |  |  |  |  |  |
| *DRB1*13:03* | 0.0096 |  |  |  |  |  |  |  |  |
| *DRB1*15:02* | 0.0096 |  |  |  |  |  |  |  |  |
| *DRB1*11:03* | 0.0096 |  |  |  |  |  |  |  |  |
| *DRB1*09:01* | 0.0048 |  |  |  |  |  |  |  |  |
| *DRB1*04:11* | 0.0048 |  |  |  |  |  |  |  |  |
| *DRB1*04:07* | 0.0048 |  |  |  |  |  |  |  |  |

| HLA-E | AF | HLA-G | AF |
| --- | --- | --- | --- |
| *E*01:01* | 0.5464 | *G*01:01* | 0.7394 |
| *E*01:03* | 0.4536 | *G*01:04* | 0.1223 |
|  |  | *G*01:06* | 0.0691 |
|  |  | *G*01:03* | 0.0585 |
|  |  | *G*01:05N* | 0.0053 |
|  |  | *G*01:08* | 0.0053 |

**Table S5.** Frequencies of HLA alleles in the donors stimulated with EBV peptide pool. Allele Frequency (AF).

| HLA-A | AF | HLA-B | AF | HLA-C | AF |
| --- | --- | --- | --- | --- | --- |
| *A*02:01* | 0.2528 | *B*08:01* | 0.1193 | *C*07:01* | 0.1847 |
| *A*01:01* | 0.1335 | *B*44:03* | 0.0938 | *C*04:01* | 0.1023 |
| *A*24:02* | 0.1278 | *B*07:02* | 0.0852 | *C*07:02* | 0.0966 |
| *A*03:01* | 0.0824 | *B*18:01* | 0.0767 | *C*05:01* | 0.0966 |
| *A*29:02* | 0.0739 | *B*51:01* | 0.0597 | *C*06:02* | 0.0881 |
| *A*11:01* | 0.0710 | *B*44:02* | 0.0455 | *C*16:01* | 0.0824 |
| *A*30:02* | 0.0455 | *B*14:02* | 0.0398 | *C*12:03* | 0.0682 |
| *A*26:01* | 0.0341 | *B*49:01* | 0.0398 | *C*02:02* | 0.0455 |
| *A*32:01* | 0.0284 | *B*57:01* | 0.0369 | *C*08:02* | 0.0426 |
| *A*23:01* | 0.0256 | *B*35:01* | 0.0341 | *C*03:04* | 0.0284 |
| *A*02:05* | 0.0170 | *B*38:01* | 0.0341 | *C*01:02* | 0.0256 |
| *A*68:01* | 0.0142 | *B*15:01* | 0.0284 | *C*03:03* | 0.0227 |
| *A*31:01* | 0.0142 | *B*40:01* | 0.0284 | *C*15:02* | 0.0199 |
| *A*33:01* | 0.0142 | *B*35:03* | 0.0227 | *C*15:05* | 0.0170 |
| *A*29:01* | 0.0085 | *B*13:02* | 0.0227 | *C*07:18* | 0.0170 |
| *A*69:01* | 0.0085 | *B*40:02* | 0.0199 | *C*12:02* | 0.0085 |
| *A*25:01* | 0.0085 | *B*27:05* | 0.0199 | *C*07:04* | 0.0085 |
| *A*34:02* | 0.0057 | *B*58:01* | 0.0199 | *C*14:02* | 0.0057 |
| *A*68:02* | 0.0057 | *B*37:01* | 0.0170 | *C*16:02* | 0.0057 |
| *A*01:02* | 0.0057 | *B*45:01* | 0.0142 | *C*17:03* | 0.0057 |
| *A*30:04* | 0.0057 | *B*39:01* | 0.0142 | *C*17:01* | 0.0057 |
| *A*02:02* | 0.0057 | *B*50:01* | 0.0142 | *C*03:05* | 0.0053 |
| *A*80:01* | 0.0028 | *B*14:01* | 0.0114 | *C*03:02* | 0.0028 |
| *A*02:06* | 0.0028 | *B*07:05* | 0.0085 | *C*15:13* | 0.0028 |
| *A*30:01* | 0.0028 | *B*55:01* | 0.0085 | *C*03:07* | 0.0028 |
| *A*24:03* | 0.0028 | *B*52:01* | 0.0085 | *C*15:04* | 0.0028 |
|  |  | *B*35:08* | 0.0085 | *C*04:03* | 0.0028 |
|  |  | *B*67:01* | 0.0057 | *C*16:04* | 0.0028 |
|  |  | *B*35:02* | 0.0057 |  |  |
|  |  | *B*15:17* | 0.0057 |  |  |
|  |  | *B*41:02* | 0.0057 |  |  |
|  |  | *B*15:18* | 0.0057 |  |  |
|  |  | *B*41:01* | 0.0057 |  |  |
|  |  | *B*56:01* | 0.0028 |  |  |
|  |  | *B*53:01* | 0.0028 |  |  |
|  |  | *B*51:05* | 0.0028 |  |  |
|  |  | *B*57:03* | 0.0028 |  |  |
|  |  | *B*39:06* | 0.0028 |  |  |
|  |  | *B*47:01* | 0.0028 |  |  |
|  |  | *B*44:05* | 0.0028 |  |  |
|  |  | *B*27:07* | 0.0028 |  |  |
|  |  | *B*07:06* | 0.0028 |  |  |
|  |  | *B*44:302* | 0.0028 |  |  |
|  |  | *B*35:43* | 0.0028 |  |  |
|  |  | *B*13:01* | 0.0028 |  |  |

| HLA-DRB1 | AF | HLA-DQA1 | AF | HLA-DQB1 | AF | HLA-DPA1 | AF | HLA-DPB1 | AF |
| --- | --- | --- | --- | --- | --- | --- | --- | --- | --- |
| *DRB1*07:01* | 0.1847 | *DQA1*05:01P* | 0.3101 | *DQB1*02:01* | 0.1790 | *DPA1*01:03P* | 0.7237 | *DPB1*04:01P* | 0.3457 |
| *DRB1*03:01* | 0.1818 | *DQA1*01:01P* | 0.1772 | *DQB1*05:01* | 0.1591 | *DPA1*02:01P* | 0.2500 | *DPB1*02:01P* | 0.1389 |
| *DRB1*15:01* | 0.0881 | *DQA1*02:01P* | 0.1709 | *DQB1*02:02* | 0.1562 | *DPA1*02:02P* | 0.0263 | *DPB1*03:01P* | 0.0895 |
| *DRB1*01:01* | 0.0881 | *DQA1*01:02P* | 0.1519 | *DQB1*03:01* | 0.1335 |  |  | *DPB1*04:02P* | 0.0864 |
| *DRB1*13:01* | 0.0540 | *DQA1*03:01P* | 0.1044 | *DQB1*06:02* | 0.0852 |  |  | *DPB1*01:01P* | 0.0710 |
| *DRB1*13:02* | 0.0426 | *DQA1*01:03P* | 0.0601 | *DQB1*03:02* | 0.0739 |  |  | *DPB1*11:01P* | 0.0525 |
| *DRB1*11:01* | 0.0426 | *DQA1*04:01P* | 0.0253 | *DQB1*06:03* | 0.0568 |  |  | *DPB1*17:01P* | 0.0401 |
| *DRB1*01:02* | 0.0369 |  |  | *DQB1*06:04* | 0.0369 |  |  | *DPB1*02:02P* | 0.0370 |
| *DRB1*11:04* | 0.0312 |  |  | *DQB1*03:03* | 0.0284 |  |  | *DPB1*10:01P* | 0.0278 |
| *DRB1*04:02* | 0.0256 |  |  | *DQB1*04:02* | 0.0284 |  |  | *DPB1*14:01P* | 0.0247 |
| *DRB1*04:04* | 0.0227 |  |  | *DQB1*05:02* | 0.0170 |  |  | *DPB1*13:01P* | 0.0247 |
| *DRB1*08:01* | 0.0227 |  |  | *DQB1*03:19* | 0.0142 |  |  | *DPB1*05:01P* | 0.0185 |
| *DRB1*12:01* | 0.0199 |  |  | *DQB1*05:03* | 0.0114 |  |  | *DPB1*06:01P* | 0.0154 |
| *DRB1*14:54* | 0.0170 |  |  | *DQB1*06:01* | 0.0085 |  |  | *DPB1*20:01P* | 0.0062 |
| *DRB1*10:01* | 0.0170 |  |  | *DQB1*06:09* | 0.0085 |  |  | *DPB1*09:01P* | 0.0062 |
| *DRB1*04:01* | 0.0170 |  |  | *DQB1*03:289* | 0.0028 |  |  | *DPB1*15:01P* | 0.0062 |
| *DRB1*16:01* | 0.0170 |  |  |  |  |  |  | *DPB1*19:01P* | 0.0031 |
| *DRB1*11:02* | 0.0142 |  |  |  |  |  |  | *DPB1*39:01P* | 0.0031 |
| *DRB1*04:05* | 0.0085 |  |  |  |  |  |  | *DPB1*16:01P* | 0.0031 |
| *DRB1*13:03* | 0.0085 |  |  |  |  |  |  |  |  |
| *DRB1*01:03* | 0.0085 |  |  |  |  |  |  |  |  |
| *DRB1*04:07* | 0.0085 |  |  |  |  |  |  |  |  |
| *DRB1*04:03* | 0.0085 |  |  |  |  |  |  |  |  |
| *DRB1*15:02* | 0.0057 |  |  |  |  |  |  |  |  |
| *DRB1*11:03* | 0.0057 |  |  |  |  |  |  |  |  |
| *DRB1*03:02* | 0.0028 |  |  |  |  |  |  |  |  |
| *DRB1*09:01* | 0.0028 |  |  |  |  |  |  |  |  |
| *DRB1*04:11* | 0.0028 |  |  |  |  |  |  |  |  |
| *DRB1*12:03* | 0.0028 |  |  |  |  |  |  |  |  |
| *DRB1*11:14* | 0.0028 |  |  |  |  |  |  |  |  |

| HLA-E | AF | HLA-G | AF |
| --- | --- | --- | --- |
| *E*01:01* | 0.5394 | *G*01:01* | 0.7327 |
| *E*01:03* | 0.4545 | *G*01:04* | 0.1384 |
| *E*01:06* | 0.0061 | *G*01:06* | 0.0629 |
|  |  | *G*01:03* | 0.0597 |
|  |  | *G*01:05N* | 0.0031 |
|  |  | *G*01:08* | 0.0031 |

**Table S6.** Fisher’s exact test analysis of IFN-γ ELISpot data stimulated with CMV pp65 by HLA

| Category | HLA | Allele | *p* value | Category with allele. n (%) | Category Others. n (%) | No Category with allele. n (%) | No Category others. n (%) |
| --- | --- | --- | --- | --- | --- | --- | --- |
| High Responders (HR) | *HLA-A* | *A*23:01* | 0.0318 | 4 (5.88%) | 64 (94.12%) | 3 (1.10%) | 269 (98.90%) |
|  |  | *A*24:02* | 0.0322 | 12 (17.65%) | 56 (82.35%) | 23 (8.71%) | 241 (91.29%) |
|  |  | *A*02:01* | 0.0004 | 24 (35.29%) | 44 (64.71%) | 39 (15.48%) | 213 (84.52%) |
|  | *HLA-B* | *B*41:02* | 0.0387 | 2 (2.70%) | 72 (97.30%) | 0 (0%) | 300 (100%) |
|  |  | *B*50:01* | 0.0259 | 3 (45%) | 71 (95.95%) | 1 (0.33%) | 298 (99.67%) |
|  |  | *B*37:01* | 0.0062 | 4 (5.41%) | 70 (94.59%) | 1 (0.34%) | 297 (99.66%) |
|  |  | *B*44:02* | 0.0180 | 5 (6.76%) | 69 (93.24%) | 4 (1.35%) | 293 (98.65%) |
|  |  | *B*40:01* | 0.0305 | 5 (6.76%) | 69 (93.24%) | 5 (1.68%) | 292 (98.32%) |
|  | *HLA-C* | *C*17:03* | 0.0375 | 2 (2.94%) | 66 (976%) | 0 (0%) | 281 (100%) |
|  |  | *C*03:04* | 0.0285 | 5 (7.35%) | 63 (92.65%) | 5 (1.80%) | 273 (98.20%) |
|  |  | *C*05:01* | 0.0266 | 8 (11.76%) | 60 (88.24%) | 12 (4.36%) | 263 (95.64%) |
|  |  | *C*06:02* | 0.0058 | 11 (16.18%) | 57 (83.82%) | 15 (5.51%) | 257 (94.49%) |
|  | *HLA-DRB1* | *DRB1*08:01* | 0.0296 | 3 (4.17%) | 69 (95.83%) | 1 (0.36%) | 273 (99.64%) |
|  |  | *DRB1*11:01* | 0.0223 | 6 (8.33%) | 66 (91.67%) | 6 (2.21%) | 265 (97.79%) |
|  |  | *DRB1*07:01* | 0.0002 | 17 (23.61%) | 55 (76.39%) | 18 (6.92%) | 242 (93.08%) |
|  | *HLA-DQB1* | *DQB1*02:02* | 0.0006 | 16 (24.24%) | 50 (75.76%) | 21 (8.14%) | 237 (91.86%) |
|  | *HLA-DQA1* | *DQB1*02:01* | 0.0001 | 16 (27.59%) | 42 (72.41%) | 15 (7.28%) | 191 (92.72%) |
|  | *HLA-DPB1* | *DPB1*17:01P* | 0.0026 | 6 (9.38%) | 58 (90.62%) | 3 (1.17%) | 253 (98.83%) |
|  |  | *DPB1*04:01P* | 0.0338 | 19 (29.69%) | 45 (70.31%) | 44.0 (18.11%) | 199 (81.89%) |
|  | *HLA-G* | *G *01:04* | 0.0226 | 12 (42.86%) | 16 (57.14%) | 23 (21.50%) | 84 (78.50%) |
| Medium Responders (MR) | *HLA-A* | *A*02:05* | 0.0135 | 138 (97.18%) | 0 (0%) | 272 (100%) | 4 (2.82%) |
|  |  | *A*11:01* | 0.0052 | 131 (92.25%) | 5 (1.89%) | 260 (98.11%) | 11 (7.75%) |
|  |  | *A*03:01* | 0.0246 | 128 (90.14%) | 10 (3.82%) | 252 (96.18%) | 14 (9.86%) |
|  |  | *A*01:01* | 0.0065 | 124 (87.32%) | 13 (54%) | 245 (94.96%) | 18 (12.68%) |
|  |  | *A*02:01* | 0.0340 | 112 (78.87%) | 33 (13.41%) | 213 (86.59%) | 30 (21.13%) |
|  | *HLA-B* | *B*52:01* | 0.0130 | 5 (3.25%) | 149 (96.75%) | 0 (0%) | 297 (100%) |
|  |  | *B*35:01* | 0.0219 | 7 (4.55%) | 147 (95.45%) | 3 (12%) | 292 (98.98%) |
|  |  | *B*51:01* | 0.0049 | 11 (7.14%) | 143 (92.86%) | 5 (1.72%) | 286 (98.28%) |
|  |  | *B*07:02* | 0.0011 | 19 (12.34%) | 135 (87.66%) | 11 (3.89%) | 272 (96.11%) |
|  | *HLA-C* | *C*12:02* | 0.0138 | 4 (2.72%) | 143 (97.28%) | 0 (0%) | 279 (100%) |
|  |  | *C*15:02* | 0.0204 | (3.40%) | 142 (96.60%) | 1 (0.36%) | 277 (99.64%) |
|  |  | *C*04:01* | 0.0004 | 19 (12.93%) | 128 (877%) | 9 (3.41%) | 255 (96.59%) |
|  |  | *C*07:02* | 0.0013 | 19 (12.93%) | 128 (877%) | 11 (4.17%) | 253 (95.83%) |
|  | *HLA-DPA1* | *DPA1*02:01P* | 0.0452 | 25 (43.86%) | 32 (56.14%) | 31 (29.25%) | 75 (70.75%) |
|  | *HLA-DPB1* | *DPB1*06:01P* | 0.0117 | 4 (3.12%) | 124 (96.88%) | 0 (0%) | 258 (100%) |
|  |  | *DPB1*04:02P* | 0.0000 | 22 (17.19%) | 106 (82.81%) | 8 (3.33%) | 232 (96.67%) |
|  |  | *DPB1*03:01P* | 0.0287 | 16 (12.50%) | 112 (87.50%) | 15 (6.10%) | 231 (93.90%) |
|  |  | *DPB1*02:01P* | 0.0075 | 21 (16.41%) | 107 (83.59%) | 18 (7.47%) | 223 (92.53%) |
|  |  | *DPB1*04:01P* | 0.0107 | 31 (24.22%) | 97 (75.78%) | 32 (13.85%) | 199 (86.15%) |
|  | *HLA-E* | *E*01:03* | 0.0028 | 44 (48.89%) | 46 (51.11%) | 33 (28.95%) | 81 (715%) |
|  |  | *E*01:01* | 0.0042 | 44 (48.89%) | 46 (51.11%) | 34 (29.82%) | 80 (70.18%) |
|  | *HLA-DRB1* | *DRB1*15:02* | 0.0403 | 3 (28%) | 141 (97.92%) | 0 (0%) | 274 (100%) |
|  |  | *DRB1*11:04* | 0.0495 | 6 (4.17%) | 138 (95.83%) | 3 (1.11%) | 268 (98.89%) |
|  |  | *DRB1*04:04* | 0.0495 | 6 (4.17%) | 138 (95.83%) | 3 (1.11%) | 268 (98.89%) |
|  |  | *DRB1*01:01* | 0.0093 | 14 (9.72%) | 130 (90.28%) | 9 (3.42%) | 254 (96.58%) |
|  | *HLA-DQB1* | *DQB1*06:03* | 0.0206 | 10 (74%) | 132 (92.96%) | 6 (2.27%) | 258 (97.73%) |
|  |  | *DQB1*03:02* | 0.0262 | 13 (9.15%) | 129 (90.85%) | 10 (3.83%) | 251 (96.17%) |
|  |  | *DQB1*05:01* | 0.0003 | 25 (17.61%) | 117 (82.39%) | 15 (62%) | 234 (93.98%) |
|  | *HLA-DQA1* | *DQA1*03:01P* | 0.0142 | 16 (13.79%) | 100 (86.21%) | 12 (5.83%) | 194 (94.17%) |
|  |  | *DQA1*01:01P* | 0.0008 | 24 (20.69%) | 92 (79.31%) | 15 (7.58%) | 183 (92.42%) |
|  |  | *DQA1*01:02P* | 0.0195 | 22 (18.97%) | 94 (813%) | 20 (10%) | 180 (90%) |
| Low Responders (LR) | *HLA-A* | *A*26:01* | 0.0160 | 6 (99%) | 60 (90.91%) | 6 (2.22%) | 264 (97.78%) |
|  |  | *A*01:01* | 0.0255 | 11 (16.67%) | 55 (83.33%) | 20 (7.55%) | 245 (92.45%) |
|  | *HLA-B* | *B*57:01* | 0.0177 | 6 (8.11%) | 68 (91.89%) | 6 (23%) | 290 (97.97%) |
|  |  | *B*08:01* | 0.0009 | 15 (20.27%) | 59 (79.73%) | 19 (6.62%) | 268 (93.38%) |
|  | *HLA-C* | *C*07:01* | 0.0059 | 16 (23.53%) | 52 (76.47%) | 28 (10.49%) | 239 (89.51%) |
|  | *HLA-DPB1* | *DPB1*01:01P* | 0.0128 | 10 (14.29%) | 60 (85.71%) | 13 (5.16%) | 239 (94.84%) |
|  | *HLA-DRB1* | *DRB1*03:01* | 0.0027 | 17 (27.87%) | 44 (72.13%) | 31 (11.92%) | 229 (88.08%) |
|  | *HLA-DQB1* | *DQB1*02:01* | 0.0050 | 17 (25.76%) | 49 (74.24%) | 30 (11.67%) | 227 (88.33%) |
|  | *HLA-DQA1* | *DQA1*05:01* | 0.0352 | 17 (35.42%) | 31 (64.58%) | 44 (21.46%) | 161 (78.54%) |

**Table S7.** Fisher’s exact test analysis of IFN-γ ELISpot data stimulated with CMV IE1 by HLA

| Category | HLA | Allele | *p* value | Category with allele. n (%) | Category Others. n (%) | No Category with allele. n (%) | No Category others. n (%) |
| --- | --- | --- | --- | --- | --- | --- | --- |
| High Responders (HR) | HLA-A | *A*02:02* | 0.0400 | 2 (4.35%) | 44 (95.65%) | 0 (0%) | 182 (100%) |
|  |  | *A*24:02* | 0.0433 | 8 (17.39%) | 38 (82.61%) | 13 (7.39%) | 163 (92.61%) |
|  | HLA-B | *B*41:01* | 0.0426 | 2 (3.85%) | 50 (96.15%) | 0 (0%) | 198 (100%) |
|  |  | *B*37:01* | 0.0296 | 3 (5.77%) | 49 (94.23%) | 1 (0.51%) | 196 (99.49%) |
|  |  | *B*40:01* | 0.0117 | 5 (9.62%) | 47 (90.38%) | 3 (1.54%) | 192 (98.46%) |
|  | HLA-C | *C*17:01* | 0.0428 | 2 (4.17%) | 46 (95.83%) | 0 (0%) | 182 (100%) |
|  |  | *C*03:04* | 0.0118 | 5 (10.42%) | 43 (89.58%) | 3 (1.68%) | 176 (98.32%) |
|  |  | *C*06:02* | 0.0213 | 7 (14.58%) | 41 (85.42%) | 8 (4.52%) | 169 (95.48%) |
|  | HLA-DRB1 | *DRB1*13:02* | 0.0271 | 5 (10.87%) | 41 (89.13%) | 5 (2.65%) | 184 (97.35%) |
|  | HLA-DQB1 | *DQB1*03:03* | 0.0257 | 3 (6.52%) | 43 (93.48%) | 1 (0.54%) | 184 (99.46%) |
|  |  | *DQB1*06:04* | 0.0041 | 5 (10.87%) | 41 (89.13%) | 2 (19%) | 181 (98.91%) |
|  | HLA-DQA1 | *DQA1*02:01* | 0.0213 | 10 (25%) | 30 (75%) | 17 (10.62%) | 143 (89.38%) |
|  | HLA-DPB1 | *DPB1*02:01P* | 0.0328 | 7 (26.92%) | 19 (738%) | 15 (10.64%) | 126 (89.36%) |
| Medium Responders (MR) | HLA-A | *A*11:01* | 0.0191 | 9 (9.78%) | 83 (90.22%) | 5 (2.86%) | 170 (97.14%) |
|  |  | *A*01:01* | 0.0022 | 14 (15.22%) | 78 (84.78%) | 7 (4.12%) | 163 (95.88%) |
|  |  | *A*02:01* | 0.0226 | 21 (22.83%) | 71 (77.17%) | 20 (12.27%) | 143 (87.73%) |
|  | HLA-B | *B*14:02* | 0.0160 | 5 (5.21%) | 91 (94.79%) | 1 (0.51%) | 194 (99.49%) |
|  |  | *B*15:01* | 0.0012 | 6 (6.25%) | 90 (93.75%) | 0 (0%) | 194 (100%) |
|  |  | *B*44:02* | 0.0176 | 6 (6.25%) | 90 (93.75%) | 2 (13%) | 192 (98.97%) |
|  |  | *B*07:02* | 0.0210 | 15 (15.62%) | 81 (84.38%) | 13 (73%) | 172 (92.97%) |
|  | HLA-C | *C*03:03* | 0.0414 | 4 (4.55%) | 84 (95.45%) | 1 (0.56%) | 179 (99.44%) |
|  |  | *C*07:02* | 0.0228 | 15 (175%) | 73 (82.95%) | 12 (7.10%) | 157 (92.90%) |
|  | HLA-DPA1 | *DPA1*01:03P* | 0.0074 | 26 (50.98%) | 25 (492%) | 17 (26.98%) | 46 (732%) |
|  | HLA-DPB1 | *DPB1*14:01P* | 0.0040 | 5 (5.95%) | 79 (94.05%) | 0 (0%) | 165 (100%) |
|  |  | *DPB1*03:01P* | 0.0470 | 10 (11.90%) | 74 (88.10%) | 8 (50%) | 152 (950%) |
|  |  | *DPB1*04:01P* | 0.0274 | 22 (26.19%) | 62 (73.81%) | 22 (14.86%) | 126 (85.14%) |
|  | HLA-E | *E*01:03* | 0.0470 | 25 (50%) | 25 (50%) | 25 (33.33%) | 50 (66.67%) |
|  |  | *E*01:01* | 0.0470 | 25 (50%) | 25 (50%) | 25 (33.33%) | 50 (66.67%) |
|  | HLA-DRB1 | *DRB1*01:02* | 0.0177 | 5 (5.21%) | 91 (94.79%) | 1 (0.53%) | 188 (99.47%) |
|  |  | *DRB1*13:01* | 0.0428 | 6 (6.25%) | 90 (93.75%) | 3 (1.60%) | 185 (98.40%) |
|  |  | *DRB1*01:01* | 0.0192 | 8 (8.33%) | 88 (91.67%) | 4 (2.15%) | 182 (97.85%) |
|  |  | *DRB1*15:01* | 0.0020 | 15 (15.62%) | 81 (84.38%) | 8 (4.47%) | 171 (95.53%) |
|  | HLA-DQB1 | *DQB1*06:02* | 0.0015 | 15 (16.67%) | 75 (83.33%) | 8 (4.62%) | 165 (95.38%) |
|  |  | *DQB1*05:01* | 0.0029 | 15 (16.67%) | 75 (83.33%) | 9 (5.20%) | 164 (94.80%) |
|  |  | *DQB1*02:02* | 0.0237 | 14 (15.56%) | 76 (84.44%) | 12 (6.90%) | 162 (93.10%) |
|  | HLA-DQA1 | *DQA1*01:01P* | 0.0042 | 14 (17.50%) | 66 (82.50%) | 7 (5.22%) | 127 (94.78%) |
|  |  | *DQA1*01:02P* | 0.0121 | 18 (22.50%) | 62 (77.50%) | 13 (10%) | 117 (90%) |
|  |  | *DQA1*05:02P* | 0.0238 | 22 (27.50%) | 58 (72.50%) | 19 (158%) | 107 (84.92%) |
| Low Responders (LR) | HLA-A | *A*02:05* | 0.0400 | 2 (4.35%) | 44 (95.65%) | 0 (0%) | 182 (100%) |
|  |  | *A*26:01* | 0.0331 | 4 (8.70%) | 42 (91.30%) | 3 (1.67%) | 177 (98.33%) |
|  | HLA-B | *B*38:01* | 0.0188 | 4 (7.69%) | 48 (92.31%) | 2 (12%) | 194 (98.98%) |
|  |  | *B*49:01* | 0.0188 | 4 (7.69%) | 48 (92.31%) | 2 (12%) | 194 (98.98%) |
|  |  | *B*51:01* | 0.0220 | 5 (9.62%) | 47 (90.38%) | 4 (25%) | 191 (97.95%) |
|  | HLA-C | *C*02:02* | 0.0373 | 4 (8.33%) | 44 (91.67%) | 3 (1.67%) | 177 (98.33%) |
|  |  | *C*12:03* | 0.0374 | 5 (10.42%) | 43 (89.58%) | 5 (2.79%) | 174 (97.21%) |
|  |  | *C*07:01* | 0.0293 | 10 (20.83%) | 38 (79.17%) | 16 (9.20%) | 158 (90.80%) |
|  | HLA-DPB1 | *DPB1*04:02P* | 0.0250 | 7 (15.22%) | 39 (84.78%) | 8 (4.91%) | 155 (95.09%) |

**Table S8.** Fisher’s exact test analysis of IFN-γ ELISpot data stimulated with EBV by HLA.

| Category | HLA | Allele | *p* value | Category with allele. n (%) | Category Others. n (%) | No Category with allele. n (%) | No Category others. n (%) |
| --- | --- | --- | --- | --- | --- | --- | --- |
| High Responders (HR) | HLA-A | *A*23:01* | 0.0193 | 5 (6.76%) | 69 (93.24%) | 4 (1.37%) | 287 (98.63%) |
|  |  | *A*11:01* | 0.0230 | 8 (10.81%) | 66 (89.19%) | 11 (3.82%) | 277 (96.18%) |
|  | HLA-B | *B*40:01* | 0.0057 | 6 (7.50%) | 74 (92.50%) | 4 (1.25%) | 316 (98.75%) |
|  |  | *B*44:02* | 0.0178 | 6 (7.50%) | 74 (92.50%) | 6 (1.88%) | 314 (98.12%) |
|  | HLA-C | *C*03:04* | 0.0051 | 6 (8.22%) | 67 (91.78%) | 4 (1.34%) | 295 (98.66%) |
|  |  | *C*05:01* | 0.0057 | 11 (157%) | 62 (84.93%) | 15 (5.10%) | 279 (94.90%) |
|  | HLA-DRB1 | *DRB1*11:03* | 0.0433 | 2 (2.44%) | 80 (97.56%) | 0 (0%) | 310 (100%) |
|  |  | *DRB1*04:04* | 0.0015 | 6 (7.32%) | 76 (92.68%) | 2 (0.65%) | 304 (99.35%) |
|  | HLA-DQB1 | *DQB1*03:02* | 0.0097 | 10 (13.51%) | 64 (86.49%) | 14 (4.70%) | 284 (95.30%) |
|  | HLA-DQA1 | *DQA1*03:01* | 0.0072 | 12 (20%) | 48 (80%) | 18 (7.63%) | 218 (92.37%) |
|  | HLA-DPB1 | *DPB1*03:01P* | 0.0356 | 10 (13.89%) | 62 (86.11%) | 17 (6.30%) | 253 (93.70%) |
| Medium Responders (MR) | HLA-A | *A*29:02* | 0.0347 | 13 (8.67%) | 137 (91.33%) | 11 (3.89%) | 272 (96.11%) |
|  |  | *A*01:01* | 0.0135 | 18 (10%) | 132 (88%) | 15 (5.40%) | 263 (94.60%) |
|  |  | *A*24:02* | 0.0015 | 22 (14.67%) | 128 (85.33%) | 15 (5.47%) | 259 (94.53%) |
|  |  | *A*02:01* | 0.0000 | 40 (26.67%) | 110 (73.33%) | 27 (10.55%) | 229 (89.45%) |
|  | HLA-B | *B*14:01* | 0.0131 | 4 (2.41%) | 162 (97.59%) | 0 (0%) | 322 (100%) |
|  |  | *B*39:01* | 0.0479 | 4 (2.41%) | 162 (97.59%) | 1 (0.31%) | 321 (99.69%) |
|  |  | *B*50:01* | 0.0479 | 4 (2.41%) | 162 (97.59%) | 1 (0.31%) | 321 (99.69%) |
|  |  | *B*35:03* | 0.0214 | 6 (3.61%) | 160 (96.39%) | 2 (0.62%) | 318 (99.38%) |
|  |  | *B*15:01* | 0.0457 | 6.9999 (4.22%) | 159 (95.78%) | 3 (0.94%) | 316 (996%) |
|  |  | *B*27:01* | 0.0019 | 10 (62%) | 156 (93.98%) | 3 (0.95%) | 313 (995%) |
|  |  | *B*51:01* | 0.0286 | 11 (6.63%) | 155 (93.37%) | 8 (2.54%) | 307 (97.46%) |
|  |  | *B*07:02* | 0.0251 | 16 (9.64%) | 150 (90.36%) | 14 (4.52%) | 296 (95.48%) |
|  |  | *B*08:01* | 0.0202 | 18 (10.84%) | 148 (89.16%) | 16 (5.19%) | 292 (94.81%) |
|  | HLA-C | *C*07:04* | 0.0380 | 3 (1.95%) | 151 (985%) | 0 (0%) | 302 (100%) |
|  |  | *C*08:02* | 0.0378 | 8 (5.19%) | 146 (94.81%) | 5 (1.68%) | 292 (98.32%) |
|  |  | *C*16:01* | 0.0059 | 17 (114%) | 137 (88.96%) | 12 (4.17%) | 276 (95.83%) |
|  |  | *C*06:02* | 0.0005 | 19 (12.34%) | 135 (87.66%) | 10 (3.50%) | 276 (96.50%) |
|  |  | *C*07:02* | 0.0240 | 16 (10.39%) | 138 (89.61%) | 14 (4.84%) | 275 (95.16%) |
|  |  | *C*04:01* | 0.0480 | 16 (10.39%) | 138 (89.61%) | 16 (5.54%) | 273 (94.46%) |
|  | HLA-DPA1 | *DPA1*02:01P* | 0.0307 | 32 (42.67%) | 43 (57.33%) | 32 (28.32%) | 81 (71.68%) |
|  |  | *DPA1*01:03P* | 0.0113 | 37 (49.33%) | 38 (50.67%) | 34 (31.48%) | 74 (68.52%) |
|  | HLA-DPB1 | *DPB1*01:01P* | 0.0113 | 14 (9.33%) | 136 (90.67%) | 9 (3.38%) | 257 (96.62%) |
|  |  | *DPB1*03:01P* | 0.0266 | 15 (10%) | 135 (90%) | 12 (4.53%) | 253 (95.47%) |
|  |  | *DPB1*04:02P* | 0.0160 | 16 (10.67%) | 134 (89.33%) | 12 (4.55%) | 252 (95.45%) |
|  |  | *DPB1*02:01P* | 0.0104 | 22 (14.67%) | 128 (85.33%) | 18 (6.98%) | 240 (932%) |
|  |  | *DPB1*04:01P* | 0.0002 | 42 (28%) | 108 (72%) | 31 (13.03%) | 207 (86.97%) |
|  | HLA-E | *E*01:03* | 0.0188 | 40 (48.78%) | 42 (51.22%) | 42 (33.33%) | 84 (66.67%) |
|  |  | *E*01:01* | 0.0188 | 40 (48.78%) | 42 (51.22%) | 42 (33.33%) | 84 (66.67%) |
|  | HLA-G | *G *01:03* | 0.0116 | 11 (15.07%) | 62 (84.93%) | 6 (4.62%) | 124 (95.38%) |
|  |  | *G *01:01* | 0.0171 | 34 (46.58%) | 39 (53.42%) | 32 (29.91%) | 75 (70.09%) |
|  | HLA-DRB1 | *DRB1*13:03* | 0.0373 | 3 (1.92%) | 153 (988%) | 0 (0%) | 309 (100%) |
|  |  | *DRB1*04:02* | 0.0435 | 6 (3.85%) | 150 (96.15%) | 3 (0.98%) | 303 (99.02%) |
|  |  | *DRB1*11:01* | 0.0090 | 10 (6.41%) | 146 (93.59%) | 5 (1.66%) | 297 (98.34%) |
|  |  | *DRB1*15:01* | 0.0150 | 16 (10.26%) | 140 (89.74%) | 13 (4.39%) | 283 (95.61%) |
|  |  | *DRB1*03:01* | 0.0025 | 30 (19.23%) | 126 (80.77%) | 26 (9.22%) | 256 (90.78%) |
|  | HLA-DQB1 | *DQB1*06:09* | 0.0383 | 3 (1.92%) | 153 (98.08%) | 0 (0%) | 305 (100%) |
|  |  | *DQB1*03:03* | 0.0213 | 7 (4.49%) | 149 (95.51%) | 3 (10%) | 298 (990%) |
|  |  | *DQB1*06:02* | 0.0273 | 15 (9.62%) | 141 (90.38%) | 13 (4.44%) | 280 (95.56%) |
|  |  | *DQB1*03:01* | 0.0015 | 22 (14.10%) | 134 (85.90%) | 15 (5.24%) | 271 (94.76%) |
|  |  | *DQB1*02:01* | 0.0020 | 30 (19.23%) | 126 (80.77%) | 25 (8.99%) | 253 (91.01%) |
|  | HLA-DQA1 | *DQA1*01:02P* | 0.0024 | 24 (195%) | 102 (80.95%) | 18 (84%) | 206 (91.96%) |
|  |  | *DQA1*05:01P* | 0.0001 | 40 (31.75%) | 86 (68.25%) | 28 (13.46%) | 180 (86.54%) |
| Low Responders (LR) | HLA-A | *A*01:02* | 0.0383 | 2 (2.78%) | 70 (97.22%) | 0 (0%) | 294 (100%) |
|  |  | *A*68:01* | 0.0061 | 4 (5.56%) | 68 (94.44%) | 1 (0.34%) | 291 (99.66%) |
|  |  | *A*26:01* | 0.0005 | 8 (11.11%) | 64 (88.89%) | 4 (1.39%) | 284 (98.61%) |
|  | HLA-B | *B*40:02* | 0.0315 | 4 (50%) | 76 (950%) | 3 (0.93%) | 319 (997%) |
|  |  | *B*49:01* | 0.0007 | 7 (8.75%) | 73 (91.25%) | 3 (0.94%) | 316 (996%) |
|  | HLA-C | *C*07:01* | 0.0133 | 16 (20.51%) | 62 (79.49%) | 29 (103%) | 260 (89.97%) |
|  | HLA-DPB1 | *DPB1*15:01P* | 0.0294 | 2 (3.45%) | 56 (96.55%) | 0 (0%) | 278 (100%) |
|  |  | *DPB1*13:01P* | 0.0098 | 5 (8.62%) | 53 (91.38%) | 4 (1.45%) | 271 (98.55%) |
|  | HLA-DRB1 | *DRB1*01:01* | 0.0382 | 10 (13.51%) | 64 (86.49%) | 19 (6.29%) | 283 (93.71%) |
|  | HLA-DQB1 | *DQB1*02:02* | 0.0413 | 14 (17.95%) | 64 (825%) | 29 (9.86%) | 265 (90.14%) |
|  |  | *DQB1*05:01* | 0.0069 | 18.0 (23.08%) | 60.0 (76.92%) | 32.0 (11.03%) | 258.0 (88.97%) |
|  | HLA-DQA1 | *DQA1*01:01* | 0.0019 | 18.0 (29.03%) | 44.0 (70.97%) | 28.0 (12.17%) | 202.0 (87.83%) |

**Table S9.** Bootstrap analysis significant *p* values, and adjusted *p* values using the Benjamini–Hochberg false-discovery rate method within each antigen-specific locus-stratified from Mann-Whitney (M-W) test or bootstrap analysis.

| Peptide | HLA | Allele | Bootstrap  *p* value | M-W  Adj *p* value | Bootstrap  Adj *p* value |
| --- | --- | --- | --- | --- | --- |
| CMV pp65 | HLA-A | *A*01:01* | 0.0006 | **0.0086** | **0.0078** |
|  |  | *A*02:01* | 1E-6 | **0.0023** | **<0.0002** |
|  |  | *A*23:01* | 0.0238 | 0.2309 | 0.2063 |
|  | HLA-B | *B*08:01* | 0.0022 | 0.0849 | **0.0429** |
|  |  | *B*35:08* | 0.0172 | 0.2798 | 0.1978 |
|  |  | *B*37:01* | 0.0434 | 0.3128 | 0.2990 |
|  |  | *B*40:01* | 0.0296 | 0.2798 | 0.2723 |
|  |  | *B*40:02* | 0.0472 | 0.3128 | 0.2990 |
|  |  | *B*44:02* | 0.0028 | 0.0849 | **0.0429** |
|  |  | *B*50:01* | 0.0028 | 0.0869 | **0.0429** |
|  | HLA-C | *C*03:04* | 0.0296 | 0.4105 | 0.3762 |
|  |  | *C*03:07* | 0.0418 | 0.6266 | 0.3762 |
|  |  | *C*07:01* | 0.0044 | 0.0710 | 0.1188 |
|  | HLA-DRB1 | *DRB1*03:01* | 0.0088 | 0.2176 | 0.1696 |
|  |  | *DRB1*08:01* | 0.0106 | 0.2176 | 0.1696 |
|  | HLA-DQB1 | *DQB1*02:01* | 0.0118 | 0.2136 | 0.1888 |
|  |  | *DQB1*04:02* | 0.0322 | 0.3229 | 0.2576 |
|  | HLA-DQA1 | *DQA1*02:01* | 0.007 | 0.0651 | 0.0630 |
|  | HLA-DPB1 | *DPB1*46:01P* | 0.0064 | 0.3666 | 0.1408 |
|  | HLA-G | *G*01:06* | 0.0122 | 0.0681 | 0.0732 |
| CMV IE1 | HLA-A | *A*01:01* | 0.043 | 0.4106 | 0.2795 |
|  |  | *A*24:02* | 0.0282 | 0.3833 | 0.2444 |
|  |  | *A*26:01* | 0.0096 | 0.3078 | 0.2444 |
|  | HLA-B | *B*37:01* | 0.0108 | 0.4414 | 0.3824 |
|  |  | *B*38:01* | 0.0408 | 0.5053 | 0.3824 |
|  |  | *B*41:01* | 0.0438 | 0.5053 | 0.3824 |
|  |  | *B*49:01* | 0.0478 | 0.5053 | 0.3824 |
|  |  | *B*51:01* | 0.022 | 0.4414 | 0.3824 |
|  | HLA-C | *C*07:01* | 0.0186 | 0.2778 | 0.3000 |
|  | HLA-DPB1 | *DPB1*01:01P* | 0.0438 | 0.1661 | 0.1643 |
|  |  | *DPB1*02:01P* | 0.0258 | 0.1661 | 0.1643 |
|  |  | *DPB1*05:01P* | 0.012 | 0.1661 | 0.1643 |
|  |  | *DPB1*17:01P* | 0.0402 | 0.1661 | 0.1643 |
|  | HLA-G | *G*01:06* | 0.0332 | 0.2451 | 0.1992 |
|  | HLA-DRB1 | *DRB1*04:02* | 0.0416 | 0.6113 | 0.6084 |
|  | HLA-DQB1 | *DQB1*03:02* | 0.0466 | 0.4116 | 0.4149 |
| EBV Consensus | HLA-A | *A*01:02* | 0.0204 | 0.2657 | 0.1326 |
|  |  | *A*11:01* | 0.0016 | **0.0340** | **0.0208** |
|  |  | *A*26:01* | 0.0102 | 0.0902 | 0.0884 |
|  |  | *A*68:01* | 0.0006 | **0.0340** | **0.0156** |
|  | HLA-B | *B*35:01* | 0.0084 | 0.2084 | 0.1932 |
|  |  | *B*40:01* | 0.0274 | 0.3595 | 0.3151 |
|  |  | *B*45:01* | 0.0134 | 0.2906 | 0.2055 |
|  |  | *B*49:01* | 0.0012 | 0.0986 | 0.0552 |
|  | HLA-C | *C*03:04* | 0.0286 | 0.5502 | 0.5201 |
|  | HLA-DPB1 | *DPB1*06:01P* | 0.0326 | 0.4117 | 0.3423 |
|  |  | *DPB1*15:01P* | 0.0264 | 0.4117 | 0.3423 |
|  | HLA-DRB1 | *DRB1*04:04* | 0.008591 | 0.2835 | 0.2574 |
|  | HLA-DQB1 | *DQB1*03:02* | 0.0368 | 0.3993 | 0.3536 |
|  |  | *DQB1*05:01* | 0.0442 | 0.3993 | 0.3536 |
|  | HLA-DQA1 | *DQA1*01:01* | 0.005 | **0.0447** | **0.0450** |
|  |  | *DQA1*03:01* | 0.0326 | 0.1501 | 0.1467 |

**Table S10.** Linkage disequilibrium of locus pairs with statistically significant differences in Fig. 3 and Fig. 4. Haplotypes with standardized residuals (Stdres) greater than 1.96 are considered significant as described in Nunes JM, et al. [13].

| Pp65 Haplotype | Observed | Expected | Stdres |
| --- | --- | --- | --- |
| *B*41:02~C*17:03* | 0.0045 | 2.03E-05 | 20.97627423 |
| *B*40:01~C*03:04* | 0.0248 | 0.00067208 | 19.60446044 |
| *DRB1*07:01~DQA1*02:01P* | 0.1914 | 0.03049878 | 19.11541665 |
| *DRB1*08:01~DQB1*04:02* | 0.0203 | 0.00050592 | 18.53855993 |
| *DQB1*02:02~DQA1*02:01P* | 0.1701 | 0.02750124 | 17.86798685 |
| *DRB1*03:01~DQB1*02:01* | 0.214 | 0.0464808 | 15.98762671 |
| *DRB1*07:01~DQB1*02:02* | 0.1689 | 0.03334482 | 15.37903926 |
| *B*08:01~C*07:01* | 0.1149 | 0.02146443 | 13.29328613 |
| *A*24:02~G*01:04* | 0.0979 | 0.01650108 | 13.24161286 |
| *A*01:01~G*01:06* | 0.0627 | 0.00787518 | 12.9664656 |
| *DQB1*02:01~DQA1*05:01P* | 0.214 | 0.0713262 | 10.8478274 |
| *DRB1*03:01~DQA1*05:01P* | 0.2135 | 0.07239276 | 10.64327491 |
| *B*08:01~DQB1*02:01* | 0.099 | 0.0250594 | 9.718029194 |
| *B*08:01~DRB1*03:01* | 0.0989 | 0.02543412 | 9.582402596 |
| *B*44:02~C*05:01* | 0.0327 | 0.0043983 | 8.972312651 |
| *A*23:01~G*01:04* | 0.0293 | 0.0043533 | 7.94964839 |
| *B*37:01~C*06:02* | 0.0135 | 0.0010989 | 7.878324294 |
| *B*50:01~C*06:02* | 0.0113 | 0.00091982 | 7.208503558 |
| *A*01:01~B*08:01* | 0.0569 | 0.01529326 | 7.034910771 |
| *B*08:01~DPB1*01:01P* | 0.0399 | 0.00914551 | 6.745295668 |
| *B*08:01~DQA1*05:01P* | 0.1021 | 0.03902943 | 6.594428752 |
| *C*07:01~DQB1*02:01* | 0.1022 | 0.0392262 | 6.567103216 |
| *C*06:02~DQA1*02:01P* | 0.0477 | 0.01291004 | 6.410028904 |
| *C*07:01~DRB1*03:01* | 0.1014 | 0.03981276 | 6.373075289 |
| *A*23:01~B*35:08* | 0.0045 | 0.0002142 | 6.169743859 |
| *A*01:01~C*07:01* | 0.067 | 0.02393898 | 5.793772813 |
| *C*06:02~DRB1*07:01* | 0.0495 | 0.01565322 | 5.655630938 |
| *C*07:01~DQA1*05:01P* | 0.1237 | 0.06109389 | 5.171546075 |
| *B*57:01~G*01:06* | 0.0125 | 0.00203814 | 4.877983812 |
| *C*06:02~DQB1*02:02* | 0.0372 | 0.01411476 | 4.065390915 |
| *A*01:01~DPB1*01:01P* | 0.0289 | 0.01019986 | 3.881619787 |
| *DQB1*02:01~DPB1*01:01P* | 0.0391 | 0.0167134 | 3.618155944 |
| *DRB1*03:01~DPB1*01:01P* | 0.0392 | 0.01696332 | 3.566899439 |
| *A*23:01~DQA1*02:01P* | 0.0167 | 0.0049959 | 3.480447407 |
| *A*01:01~DRB1*03:01* | 0.0554 | 0.02836632 | 3.333852912 |
| *B*50:01~DQA1*02:01P* | 0.0084 | 0.00179218 | 3.286013625 |
| *DQA1*05:01P~DPB1*01:01P* | 0.05 | 0.02603073 | 3.089411409 |
| *A*01:01~DQB1*02:01* | 0.0516 | 0.0279484 | 2.939125936 |
| *A*23:01~DPB1*17:01P* | 0.0057 | 0.00114975 | 2.826019964 |
| *B*44:02~DRB1*11:01* | 0.0068 | 0.00155925 | 2.794397606 |
| *A*01:01~DQA1*05:01P* | 0.0706 | 0.04352898 | 2.673885262 |
| *A*01:01~B*57:01* | 0.0126 | 0.00441428 | 2.590345899 |
| *B*37:01~DRB1*08:01* | 0.0023 | 0.0002754 | 2.570330319 |
| *A*02:01~B*44:02* | 0.022 | 0.00994275 | 2.535225797 |
| *B*50:01~DQB1*02:02* | 0.0072 | 0.00195942 | 2.49219013 |
| *DRB1*11:01~DPB1*04:01P* | 0.0251 | 0.01273195 | 2.294894877 |
| *A*23:01~DQB1*02:02* | 0.0135 | 0.0054621 | 2.285414242 |
| *B*50:01~DRB1*07:01* | 0.0072 | 0.00217299 | 2.269864842 |
| *B*37:01~DQB1*04:02* | 0.0023 | 0.0003348 | 2.262730354 |
| *B*35:08~G*01:04* | 0.0041 | 0.00093976 | 2.171195403 |
| *A*02:01~B*41:02* | 0.0045 | 0.00110475 | 2.151251627 |
| *A*02:01~C*17:03* | 0.0045 | 0.00110475 | 2.151251627 |
| *DQB1*02:02~DPB1*17:01P* | 0.0144 | 0.0063291 | 2.130903381 |
| *DRB1*07:01~DPB1*17:01P* | 0.0154 | 0.00701895 | 2.100507916 |
| *DQB1*04:02~G*01:04* | 0.0092 | 0.00342736 | 2.07415199 |
| *A*02:01~B*40:01* | 0.0135 | 0.0060884 | 1.995380615 |
| IE1 Haplotype | Observed | Expected | Stdres |
| *A*02:02~B*41:01* | 0.0045 | 2.03E-05 | 20.97627423 |
| *A*02:02~C*17:01* | 0.0045 | 2.03E-05 | 20.97627423 |
| *B*41:01~C*17:01* | 0.0045 | 2.03E-05 | 20.97627423 |
| *B*40:01~C*03:04* | 0.0248 | 0.00067208 | 19.60446044 |
| *DRB1*13:02~DQB1*06:04* | 0.0293 | 0.00128205 | 16.47770742 |
| *B*38:01~C*12:03* | 0.0315 | 0.00199395 | 13.90951586 |
| *A*01:01~G*01:06* | 0.0627 | 0.00787518 | 12.9664656 |
| *B*37:01~C*06:02* | 0.0135 | 0.0010989 | 7.878324294 |
| *B*49:01~C*07:01* | 0.0383 | 0.00742365 | 7.522993001 |
| *B*51:01~C*02:02* | 0.0194 | 0.00238502 | 7.332606784 |
| *C*06:02~DQB1*03:03* | 0.0158 | 0.0018315 | 6.871312906 |
| *A*26:01~B*38:01* | 0.0112 | 0.00099225 | 6.824885742 |
| *DRB1*04:02~DPB1*05:01P* | 0.0068 | 0.00041132 | 6.636259676 |
| *C*06:02~DQA1*02:01P* | 0.0477 | 0.01291004 | 6.410028904 |
| *C*03:04~DQB1*06:04* | 0.0097 | 0.00085365 | 6.377202883 |
| *B*40:01~DQB1*06:04* | 0.009 | 0.0007812 | 6.193684489 |
| *A*01:01~C*07:01* | 0.067 | 0.02393898 | 5.793772813 |
| *DQB1*03:03~DQA1*02:01P* | 0.0197 | 0.0035685 | 5.679983985 |
| *C*03:04~DRB1*13:02* | 0.0095 | 0.00110297 | 5.324706824 |
| *B*40:01~DRB1*13:02* | 0.009 | 0.00100936 | 5.297009046 |
| *B*38:01~DRB1*04:02* | 0.0068 | 0.0007119 | 4.806280617 |
| *A*02:05~B*49:01* | 0.0068 | 0.00082215 | 4.391191395 |
| *A*26:01~C*12:03* | 0.011 | 0.00199395 | 4.245563041 |
| *B*38:01~DQB1*03:02* | 0.0113 | 0.0021294 | 4.183094972 |
| *A*01:01~DPB1*01:01P* | 0.0289 | 0.01019986 | 3.881619787 |
| *A*02:02~DPB1*17:01P* | 0.0023 | 0.00016425 | 3.511183618 |
| *B*41:01~DPB1*17:01P* | 0.0023 | 0.00016425 | 3.511183618 |
| *DPB1*05:01P~G*01:06* | 0.0062 | 0.00109746 | 3.243733094 |
| *C*12:03~DRB1*04:02* | 0.0068 | 0.00143058 | 2.989178967 |
| *C*12:03~DQB1*03:02* | 0.0135 | 0.00427908 | 2.963871373 |
| *DRB1*04:02~G*01:06* | 0.0065 | 0.00136278 | 2.930290306 |
| *DQB1*06:04~DPB1*02:01P* | 0.0121 | 0.0044289 | 2.42346824 |
| *A*02:05~DPB1*05:01P* | 0.0024 | 0.00036946 | 2.225558832 |
| *A*24:02~C*07:02* | 0.0203 | 0.00999378 | 2.161448853 |
| *DQB1*03:02~DPB1*05:01P* | 0.0047 | 0.00123032 | 2.083073244 |
| *DRB1*13:02~DPB1*02:01P* | 0.013 | 0.00572242 | 2.021354248 |
| EBV Haplotype | Observed | Expected | Stdres |
| *B*40:01~C*03:04* | 0.0248 | 0.00067208 | 19.60446044 |
| *DQB1*03:02~DQA1*03:01P* | 0.0676 | 0.0067262 | 15.58732174 |
| *DQB1*05:01~DQA1*01:01P* | 0.1506 | 0.0284186 | 15.0534082 |
| *DRB1*01:01~DQB1*05:01* | 0.0859 | 0.01282284 | 13.5107228 |
| *DRB1*04:04~DQB1*03:02* | 0.0248 | 0.00168324 | 11.86259241 |
| *DRB1*01:01~DQA1*01:01P* | 0.0855 | 0.01552635 | 11.74068337 |
| *DRB1*04:04~DQA1*03:01P* | 0.0248 | 0.00247755 | 9.438075981 |
| *B*40:01~DRB1*04:04* | 0.0113 | 0.00061752 | 9.055322567 |
| *B*44:02~C*05:01* | 0.0327 | 0.0043983 | 8.972312651 |
| *C*03:04~DRB1*04:04* | 0.0113 | 0.00067479 | 8.615849619 |
| *A*26:01~DPB1*15:01P* | 0.0053 | 0.0001638 | 8.4555311 |
| *B*49:01~C*07:01* | 0.0383 | 0.00742365 | 7.522993001 |
| *DRB1*04:04~DPB1*06:01P* | 0.0073 | 0.00045318 | 6.775581897 |
| *A*01:02~B*49:01* | 0.0045 | 0.00018225 | 6.73869237 |
| *B*45:01~DPB1*15:01P* | 0.0023 | 7.02E-05 | 5.607555696 |
| *B*40:01~DQB1*03:02* | 0.0113 | 0.00167648 | 4.948366993 |
| *C*03:04~DQB1*03:02* | 0.0111 | 0.00183196 | 4.558513248 |
| *A*11:01~DQA1*03:01P* | 0.0221 | 0.0060496 | 4.335072566 |
| *C*03:04~DQA1*03:01P* | 0.0131 | 0.00269645 | 4.215902325 |
| *A*11:01~DRB1*04:04* | 0.0085 | 0.00151392 | 3.780459448 |
| *B*40:01~DQA1*03:01P* | 0.0113 | 0.0024676 | 3.741935856 |
| *B*45:01~DQA1*01:01P* | 0.0113 | 0.00250425 | 3.698972034 |
| *B*49:01~DRB1*01:01* | 0.0135 | 0.00338985 | 3.652763012 |
| *A*11:01~DPB1*03:01P* | 0.0186 | 0.0060192 | 3.406585976 |
| *B*49:01~DQA1*01:01P* | 0.02 | 0.00751275 | 3.024275082 |
| *A*11:01~B*40:01* | 0.0068 | 0.00150784 | 2.869585377 |
| *A*01:02~C*07:01* | 0.0045 | 0.00082485 | 2.695255105 |
| *A*11:01~C*03:04* | 0.0068 | 0.00164768 | 2.67238973 |
| *B*44:02~DQA1*03:01P* | 0.0118 | 0.00402975 | 2.574012242 |
| *B*45:01~DQB1*05:01* | 0.0075 | 0.0020682 | 2.514139781 |
| *DQA1*03:01P~DPB1*06:01P* | 0.0067 | 0.0018109 | 2.418686511 |
| *A*11:01~DQB1*03:02* | 0.0107 | 0.00411008 | 2.161484662 |
| *DRB1*01:01~DPB1*15:01P* | 0.0025 | 0.00043524 | 2.084981139 |

**Table S11.** Most frequent estimated HLA haplotypes among donors included in the study.

Haplotype frequency (HF).

| H | Haplotype | HF |
| --- | --- | --- |
| 1 | *A*01:01~C*07:01~B*08:01~DRB1*03:01~DQB1*02:01* | 0.0541 |
| 2 | *A*30:02~C*05:01~B*18:01~DRB1*03:01~DQB1*02:01* | 0.0446 |
| 3 | *A*29:02~C*16:01~B*44:03~DRB1*07:01~DQB1*02:02* | 0.037 |
| 4 | *A*02:01~C*07:02~B*07:02~ DRB1*15:01~DQB1*06:02* | 0.0294 |
| 5 | *A*02:01~C*07:01~B*08:01~DRB1*03:01~DQB1*02:01* | 0.017 |
| 6 | *A*02:01~C*05:01~B*44:02~DRB1*01:01~DQB1*05:01* | 0.0124 |
| 7 | *A*24:02~C*07:02~B*07:02~DRB1*15:01~DQB1*06:02* | 0.0123 |
| 8 | *A*02:01~C*16:01~B*44:03~DRB1*07:01~DQB1*02:02* | 0.0111 |

**Supplementary Figure 1.** Representative flow cytometry gating strategy for the identification and phenotypic characterization of antigen-responsive T cells. Representative plots showing the sequential gating strategy used to analyze T cell responses after peptide stimulation. Antigen-responsive cells were identified according to intracellular IFN-γ expression, with gates defined using the corresponding isotype control. CCR7 and CD45RA expression was used to describe differentiation phenotypes within CD4^+^ and CD8^+^ T cells, defining CCR7^+^CD45RA^+^ cells as naïve-like, CCR7^+^CD45RA^−^ cells as central-memory-like, CCR7^−^CD45RA^−^ cells as effector-memory-like, and CCR7^−^CD45RA^+^ cells as T_EMRA_-like. Representative plots are shown from one donor and are illustrative of the gating strategy applied across all analyzed samples.

**Supplementary Figure 2.** Thawed PBMCs stimulated with CMV (pp65 and IE1) or EBV (Consensus®) antigens, detection of cell response by IFN-γ ELISpot. Raw IFN-γ spot-forming units (SFU) of 2.5x10^4^ cells stimulated without or with viral antigens, 200 donors with pp65, 176 donors with IE1, and 189 donors with EBV. ELISpot negative control (C-). Median values marked in red.

**Supplementary Figure 3. Thawed PBMCs stimulated with CMV and EBV antigens, detection of cell response by IFN-γ ELISpot by responder donor characteristics.** IFN-γ SFU of 2.5x10^4^ cells stimulated with CMV or EBV antigens categorized by **(A)** blood group, **(B)** Rhesus D (RhD) antigen positive (+), or negative (-), or **(C)** Sex; females (F) and males (M). Negative control of each donor has been subtracted. Multiple-groups were analyzed by Kruskal-Wallis test followed by Dunn’s multiple-groups comparison post-test. Two groups were analyzed by the Mann-Whitney test. Median values marked in red. **(D)** Correlation of IFN-γ SFU of 2.5x10^4^ cells stimulated with CMV or EBV antigens, 164 with CMV pp65, 104 with CMV IE1, and 176 with EBV antigens, with donors’ age (years). Analyzed by Spearman correlation. Dashed lines distinguish high, medium, or low responders.

**Supplementary Figure 4. Significantly different IFN-γ ELISpot results by donor’s HLA.** IFN-γ spot forming units (SFU) of **(A)** 164 responder donors with CMV pp65**, (B)** 103 with CMV IE1, and **(C)** 189 responder donors with EBV antigens with numerically significant differences by donors with specific HLA allele compared to other (Oth) donors. Analyzed by Mann-Whitney test. Median marked in red.

**Supplementary Figure 5. Antigen-specific IFN-γ ELISpot responses according to HL allele copy number.** IFN-γ spot forming units (SFU) of **(A)** 164 responder donors with CMV pp65**, (B)** 103 with CMV IE1, and **(C)** 189 responder donors with EBV antigens. Donors were stratified as homozygous carriers (Hom), heterozygous carriers (Het), or non-carriers (Others) of the indicated HLA allele. Overall differences among the three groups were assessed using the Kruskal-Wallis test, with significant *p*-values shown in the corresponding panels. Pairwise post hoc comparisons were performed using Dunn’s multiple-comparison test, with significance indicated by asterisks (^*^*p*<0.05, ^**^*p*<0.005). Median marked in red.

**Supplementary Figure 6. T cell response analysis by HLA-G variants.** **(A)** IFN-γ SFU of 2.5x10^4^ cells stimulated with CMV IE1 distributed by four HLA-G 3’UTR variants, **(B)** and by HLA-G 3’UTR haplotypes. **(C)** IFN-γ SFU of 2.5x10^4^ cells stimulated with EBV consensus distributed by four HLA-G 3’UTR variants, **(D)** and by HLA-G 3’UTR haplotypes. Data analyzed by Mann Whitney test. **(E)** Percentages of memory T cell subsets, stem cell memory (SCM), central memory (CM), effector memory (EM), effector memory CD45RA^+^ (EMRA) among IFN-γ^+^ CD3^+^CD4^+^ and CD3^+^CD8^+^ T cells stimulated with CMV pp65 by HLA-G 3’UTR haplotype. Median marked in red.

Supplementary MATHERIALS AND methods

**Study participants samples**

From each donor, 20 mL of peripheral blood was collected in two heparin-coated tubes, along with one coagulation activator tube for serum, and two EDTA tubes. Table 1 shows the demographic characteristics of the donors included in this study.

**Cytomegalovirus and Epstein-Barr IgG detection**

Sera were screened for cytomegalovirus (CMV) and Epstein-Barr virus (EBV) IgG antibodies using Architect CMV and EBV Ig detection kits (Abbott Laboratories, Abbott Park, IL, USA), following manufacturer’s instructions. Results are reported in arbitrary units (AU)/mL.

**High-resolution HLA typing**

An automated protocol for genomic DNA extraction from peripheral blood was performed using a QIAsymphony DNA Mini Kit (Qiagen, Hilden, Germany). Typing for HLA-A, HLA-B, HLA-C, HLA-DRB1, HLA-DPA1, HLA-DPB1, HLA-DQA1, HLA-DQB1, HLA-E, and HLA-G genes, and 3’UTR of HLA-G was carried out by next generation sequencing (NGS). Amplicons were obtained by in-house multiplex PCR, and library preparation was performed by fragmentation using the NGSgo kit (GenDX, Utrecht, Netherlands). Sequencing was performed in a MiSeq (Illumina, San Francisco, CA, USA) using a MiSeq Reagent Kit v2 (300-cycles) (Illumina). Two-field HLA data analysis was done using NGSengine software (GenDx, versions 2.17.0 to 2.22.0) and the IMGT/HLA database (versions 3.39.0 to 3.44.1). P code nomenclature was used for HLA-DQA1, -DPB1, and -DPA1 genes. HLA-G 3’ variants were analyzed by QIAGEN CLC Genomics Workbench (Qiagen, Hilden, Germany).

**PBMCs peptide pools stimulation**

As previously described by our group [12], peripheral blood mononuclear cells (PBMCs) were isolated from heparin-coated tubes using density gradient centrifugation with Lymphoprep^TM^ medium (Stemcell Technologies, Vancouver, Canada) and cryopreserved until use.
